# Comparative efficacy and safety of multiple acupuncture therapies for post stroke cognitive impairment: a network meta-analysis of randomized controlled trials

**DOI:** 10.3389/fneur.2023.1218095

**Published:** 2023-08-10

**Authors:** Yang Liu, Lu Zhao, Fuyan Chen, Xingping Li, Jiangqin Han, Xiaowei Sun, Mingtong Bian

**Affiliations:** ^1^Department of Acupuncture, First Teaching Hospital of Tianjin University of Traditional Chinese Medicine, Tianjin, China; ^2^National Clinical Research Center for Chinese Medicine Acupuncture and Moxibustion, Tianjin, China

**Keywords:** acupuncture treatment, post-stroke cognitive impairment, cognitive rehabilitation, non-pharmacological treatment, network meta analysis

## Abstract

**Background:**

Acupuncture therapy has been widely used to treat post-stroke cognitive impairment (PSCI). However, acupuncture therapy includes multiple forms. Which acupuncture therapy provides the best treatment outcome for patients with PSCI remains controversial.

**Objective:**

We aimed to compare and evaluate the efficacy and safety of different acupuncture-related therapies for PSCI in an attempt to identify the best acupuncture therapies that can improve cognitive function and self-care in daily life for patients with PSCI, and bring new insights to clinical practice.

**Method:**

We searched eight databases including PubMed, Embase, Web of Science, Cochrane Central Register of Controlled Trials, China Biomedical Literature Database (CBM), China Science and Technology Journal (VIP) database, China National Knowledge Infrastructure (CNKI) database, and Wan fang database to find randomized controlled trials (RCTs) of acupuncture-related therapies for PSCI from the inception of the database to January 2023. Two researchers independently assessed the risk of bias in the included studies and extracted the study data. Pairwise meta-analyzes for direct comparisons were performed using Rev. Man 5.4 software. Bayesian network meta-analysis (NMA) was performed using STATA 17.0 and R4.2.4 software. The quality of evidence from the included studies was assessed using the Grading of Recommendations, Assessment, Development, and Evaluation (GRADE) system. Adverse effects (AEs) associated with acupuncture therapy were collected by reading the full text of the included studies to assess the safety of acupuncture therapy.

**Results:**

A total of 62 RCTs (3 three-arm trials and 59 two-arm trials) involving 5,073 participants were included in this study. In the paired meta-analysis, most acupuncture-related therapies had a positive effect on cognitive function and self-care of daily living in patients with PSCI compared with cognitive training. Bayesian NMA results suggested that ophthalmic acupuncture plus cognitive training (79.7%) was the best acupuncture therapy for improving MMSE scores, with scalp acupuncture plus cognitive training ranking as the second (73.7%). The MoCA results suggested that warm acupuncture plus cognitive training (86.5%) was the best acupuncture therapy. In terms of improvement in daily living self-care, scalp acupuncture plus body acupuncture (87.5%) was the best acupuncture therapy for improving MBI scores. The most common minor AEs included subcutaneous hematoma, dizziness, sleepiness, and pallor.

**Conclusion:**

According to our Bayesian NMA results, ophthalmic acupuncture plus cognitive training and warm acupuncture plus cognitive training were the most effective acupuncture treatments for improving cognitive function, while scalp acupuncture plus body acupuncture was the best acupuncture treatment for improving the performance of self-care in daily life in patients with PSCI. No serious adverse effects were found in the included studies, and acupuncture treatment appears to be safe and reliable. However, due to the low methodological quality of the included studies, our findings need to be treated with caution. High-quality studies are urgently needed to validate our findings.

**Systematic review registration:**

https://www.crd.york.ac.uk/prospero/#recordDetails, identifier: CRD42022378353.

## 1. Introduction

Post-stroke cognitive impairment (PSCI), Characterized by distractibility and impaired language, memory, and executive skills, has a serious impact on the quality of life and survival time of stroke survivors (1). The prevalence of PSCI is steadily climbing as the population ages and the number of stroke survivors continues to increase. In the latest meta-analysis involving 16 studies, approximately 53.4% of stroke survivors were reported to suffer from PSCI, with the incidence of mild and severe PSCI being 36.4 and 16.5%, respectively (2). One study based on 6,504 stroke patients evaluated outcomes from the first 3 months to 5 years after stroke and found that patients with PSCI were strongly associated with an increased risk of death, dependency, depression, and hospitalization (3). However, in many national and international guidelines for stroke treatment, few details about PSCI were mentioned. It is clear that PSCI is not receiving enough attention and is not effectively addressed.

Currently, the treatment of PSCI mainly consists of pharmacological and non-pharmacological treatments. Studies have confirmed that there is no strong evidence that pharmacological interventions, including cholinesterase inhibitors and memantine, can improve cognition or slow the progression of dementia (4). In addition, one study based on 168 patients with vascular cognitive impairment found no significant improvement in cognitive function or ADL with donepezil (5). Instead, we found side effects associated with these drugs, such as gastrointestinal reactions and liver toxicity (6). Therefore, non-pharmacological treatments such as acupuncture, cognitive training, and transcranial magnetic stimulation have gradually received widespread attention. A network meta-analysis (7) published in 2022 compared five nonpharmacological treatments for improving cognitive function and self-care in patients with PSCI, and its results indicated that acupuncture was the most effective treatment for improving MoCA scores in PSCI patients.

Acupuncture, as a basic treatment tool in Chinese medicine, has been widely used for thousands of years for the prevention and treatment of various diseases (8). Acupuncture indeed has better clinical efficacy for some hard-to-treat chronic diseases, such as chronic kidney disease and low back pain (9, 10). In recent years, studies (11–13) have continued to find that acupuncture has a better effect on improving cognitive function in patients with PSCI. Relevant animal experiments are also being conducted in an attempt to explore the potential mechanism of acupuncture for PSCI. However, there are many forms of acupuncture treatment, including scalp acupuncture, warm acupuncture, abdominal acupuncture, and auricular acupuncture, etc. To date, there is still no systematic review that comprehensively compares and evaluates the efficacy of multiple acupuncture therapies. The differences in efficacy between acupuncture therapies remain unclear. Similarly, it is not clear to PSCI patients and clinicians which acupuncture technique is the best choice. Therefore, we conducted this network meta-analysis. In this study, we included 62 RCTs that critically evaluated the efficacy of eight different acupuncture techniques for the treatment of PSCI to provide evidence for the clinical selection of appropriate treatment options.

## 2. Methods

### 2.1. Registration

The protocol for this meta-analysis was registered with the International Platform for the Registration of Systematic Review (PROSPERO) under registration number CRD42022378353. This study was conducted in strict accordance with the PRISMA Extension Statement for Reports of Systematic Reviews Incorporating Meta-Analyzes of Healthcare Intervention Networks (PRISMA-NMA) (14), as detailed in Supplementary Appendix S1.

### 2.2. Search strategy

We searched eight databases including PubMed, Embase, Cochrane Library, Web of Science, China National Knowledge Infrastructure (CNKI), China Biomedical Literature Database (CBM), China Science and Technology Journal (VIP) database, and Wan Fang database, from their inception to January, 2023, to find eligible RCTs regarding acupuncture treatment of PSCI. The language was restricted to Chinese or English. Meanwhile, the reference lists of systematic review articles were read to determine if there was any missing literature. The following terms were used in the search strategies: (acupuncture therapy, scalp acupuncture, warm acupuncture, electro-acupuncture) and (stroke, cerebrovascular accident, post-stroke cognitive impairment, and PSCI). The search strategy for each database was shown in Supplementary Appendix S2.

### 2.3. Eligibility criteria

We included studies that met the inclusion and exclusion eligibility criteria listed in Table 1.

**Table 1:** Eligibility criteria for relevant studies.

Criteria	Inclusion	Exclusion
Population	•Adults (18 years or older) who meet validated diagnostic instruments for PSCI	•Under 18 years old • Cognitive impairment caused by other diseases, such as Alzheimer’s disease (AD) and Cranial Trauma
Intervention	•Various acupuncture therapies (body acupuncture, scalp acupuncture, warm acupuncture, electro-acupuncture, abdominal acupuncture, auricular bloodletting, ophthalmic acupuncture, etc.) •Single or combined use of acupuncture therapy	•Other non-pharmacological treatments not covered by the study
Comparators	•Cognitive training •The different acupuncture treatments compared with the experimental group	•Other non-pharmacological treatments not covered by the study
Outcomes	•The Minimum Mental State Examination scale (MMSE) •The Montreal Cognitive Assessment Scale (MoCA) •The Modified Barthel Index scale (MBI) •Adverse events (AEs)	•Lack of valid outcome
Languages	Chinese and English	Other languages
Study designs	Randomized controlled trials (RCTs)	Reviews, animal trials, case reports, and conference papers.

### 2.4. Date extraction

Two investigators (YL and LZ) independently performed literature screening and data extraction and cross-checked the results. Any inconsistencies in the information extraction process can be resolved by the third investigator (FY-C). Extraction included basic characteristics (first author, year of publication, diagnostic criteria, sample size, gender, stroke type, age, duration of disease), details of the intervention (type of acupuncture, duration of treatment, periodicity, frequency), outcomes and adverse events (AEs).

### 2.5. Quality assessment

Two investigators (YL and LZ) independently assessed the quality of the included literature according to the risk of bias assessment tool (ROB2) recommended by the Cochrane Handbook. The assessments included the randomization process, deviation from the intended intervention, missing outcome data, measurement of the outcome, selection of the reported result, and overall bias. Each assessment component was categorized as low risk, high risk, and some concern. Given that the majority of acupuncture studies were published in Chinese journals, we used the Consolidated Standards for Reporting Trials (CONSORT) reporting guidelines (15) to assess the quality of included literature. The percentages for each item in the corresponding specifications were calculated and presented. In addition, the quality of evidence for each outcome indicator was assessed using the Grading of Recommendations, Assessments, Developments and Evaluations (GRADE) system (16), which resulted in a high, moderate, low, or very low level of evidence. Disagreements encountered during the assessment process could be resolved by the third investigator (FY-C).

### 2.6. Statistical analysis

Paired meta-analysis was performed using Rev. Man 5.4 software (Cochrane Collaboration, Oxford, United Kingdom). Effect sizes were calculated using mean differences (MDs) and 95% confidence intervals (CI) for continuous variables. Effect sizes were calculated using odd ratios (OR) and 95%CI for dichotomous variables. Heterogeneity between included studies was assessed according to the Q test (*p* value) and I^2^ statistic. If *p* ≥ 0.1 and I^2^ ≤ 50%, it represented acceptable heterogeneity, and a fixed-effects model was selected for meta-analysis, and conversely, a random-effects model was selected for meta-analysis.

STATA version 17.0 (Stata Corp, College Station, Texas, United States) and R version 4.2.4 (R Core Team, Vienna, Austria) were used for Bayesian framework network meta-analysis. Considering the possible heterogeneity among the included studies, we merged the data using random effects models. Given that the outcome variables chosen for this study were all continuous, mean differences (MDs) and 95% CI were selected for calculation. Markov chain Monte Carlo (MCMC) was used to calculate the model with the following parameters: four chains, 20,000 sample iterations, 5,000 burns, and a lean interval of 1. Brooks-Gelman-Rubin diagnostic plots were used to assess the convergence of the model. In addition, we also observed trajectory and density plots. The node-splitting method was used to assess the agreement between direct and indirect comparisons. *p* > 0.05 indicates the existence of the agreement. When there was a closed loop, we used the inconsistency factor (IF) to make the judgment. When the 95% CI contains 0, it indicates the existence of consistency between direct and indirect evidence. The surface under the cumulative ranking area (SUCRA) was calculated to rank each intervention probabilistically. The value of SUCRA ranged from 0 to 100%, with higher values indicating better efficacy. The following formula was used to approximate the outcome data, taking into account possible differences in baseline conditions for outcome indicators in the included studies, where the correlation coefficient R-value was 0.5.


(1) $${\bar{MDs}}_{Change}={\bar{MDs}}_{Final}-{\bar{MDs}}_{Baseline}$$

(2) $$SD_{Change}=\sqrt{\begin{array}{l} {\left(SD_{Baseline}\right)}^{2}+{\left(SD_{Final}\right)}^{2}-\\ \left(2\times R\times SD_{Baseline}\times SD_{Final}\right) \end{array}}$$

## 3. Results

### 3.1. Literature selection

**Figure 1:**
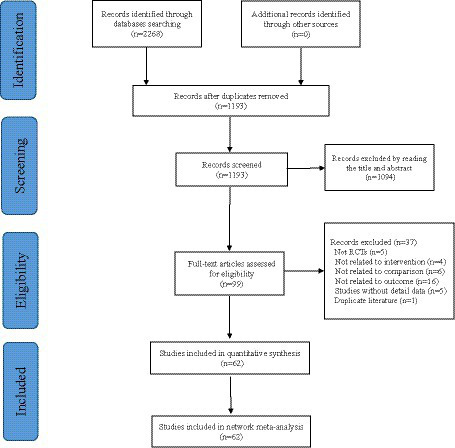
Flow chart of the literature screening process.

We initially searched for 2,268 potentially relevant articles and excluded 1,075 articles due to duplication. The 1,094 articles were excluded by reading the titles and abstracts. The remaining 99 studies were evaluated by reading the full text. 62 studies (17–78) were ultimately included in the quantitative analysis. The PRISMA flowchart of the search process is shown in Figure 1.

### 3.2. Study characteristics

All 62 included studies were conducted in China, of which 59 and 3 RCTs were published in Chinese and English, respectively. These included studies were reported between 2012 and 2022. A total of 5,073 participants were included, of which 2,593 participants were in the experimental group and 2,480 participants were in the control group. Among the 62 studies, 3 (18, 53, 55) were three-arm trials and 59 were two-arm trials. The baseline data for participants in both groups were generally similar, but 5 studies (23, 25, 36, 41, 45) did not report the mean age and 17 studies (25, 31, 33, 34, 36–38, 41, 44–47, 50, 52, 57, 58, 69) did not report the mean duration of disease. 3 studies (23, 35, 36) reported patient drop-out and reported specific reasons, and the number of drop-outs ranged from 3 to 11. Treatment duration ranged from 2 weeks to 12 weeks. In terms of treatment measures, in addition to cognitive training (CT), 13 types of acupuncture-related therapies are included, which were body acupuncture (BA), scalp acupuncture (SA), body acupuncture plus cognitive training (BA+CT), scalp acupuncture plus cognitive training (SA + CT), ophthalmic acupuncture plus cognitive training (OA + CT), warm acupuncture plus cognitive training (WA + CT), electro-acupuncture plus cognitive training (EA + CT), auricular bloodletting plus cognitive training (AB+CT), abdominal acupuncture plus cognitive training (AA+CT), scalp acupuncture plus body acupuncture (SA + BA), abdominal acupuncture plus body acupuncture (AA+BA), warm acupuncture plus scalp acupuncture (WA + SA) and scalp acupuncture plus auricular bloodletting (SA + AB). Furthermore, 47 studies (17–23, 25–29, 31, 33–36, 38, 40, 41, 43–50, 52, 53, 55–57, 59, 60, 62–67, 70, 72, 73, 75, 77, 78) reported MMSE scores, 29 studies (18, 24, 28, 30–34, 37, 39, 42, 47–49, 52, 54, 58, 61–63, 67–72, 74, 76, 77) reported MoCA scores and 20 studies (22–24, 27, 30, 32–35, 41, 42, 47–50, 54, 60, 61, 73, 74) reported MBI scores. The characteristics of the included studies are shown in Table 2.

**Table 2:** The characteristics of the included studies.

Study	Sample size (T/C)(M/F)	Stroke type (I/H)	Mean age (Year) (T/C)	Course of disease (T/C)	Treatment (T/C)	Treatment period	Outcome	Adverse effects (AEs)	Drop-out situation (T/C)
1-Bao 2021 (17)	72 (31/41)	NA	62.4 ± 3.93	40.6 ± 8.87d	SA + BA	8 weeks	①	None	None
	72 (34/38)	NA	61.6 ± 4.75	40.4 ± 10.9d	CT				
2-Jiang 2016 (18)	51 (25/26)	31/20	62.37 ± 7.89	44.22 ± 17.00d	CT	12 weeks	①②	None	None
	52 (25/27)	37/15	61.58 ± 9.71	41.12 ± 21.71d	SA				
	52 (23/29)	35/17	62.33 ± 7.22	41.13 ± 18.80d	SA + CT				
3-Jian 2020 (19)	35 (20/15)	NA	63.00 ± 7.23	2.13 ± 0.85 m	SA + CT	12 weeks	①	NA	None
	35 (17/18)	NA	65.30 ± 8.52	2.51 ± 0.46 m	CT				
4-Bai 2012 (20)	30 (16/14)	NA	59.85 ± 6.03	11.71 ± 3.65d	SA + CT	4 weeks	①	NA	None
	30 (18/12)	NA	60.39 ± 5.67	16.93 ± 2.97d	CT				
5-Pu 2018 (21)	53 (33/20)	NA	60.37 ± 5.45	5.12 ± 1.39d	SA + BA	12 weeks	①	NA	None
	53 (34/19)	NA	59.15 ± 5.29	5.33 ± 1.40d	CT				
6-Cai 2020 (22)	43 (20/23)	NA	61.35 ± 5.63	9.65 ± 3.26d	SA + BA	4 weeks	①③	NA	None
	43 (22/21)	NA	64.23 ± 5.24	10.32 ± 2.61d	CT				
7-Zeng 2018 (23)	40 (22/18)	25/15	NA	71.00 ± 42.73d	SA + CT	4 weeks	①③	NA	6/5
	40 (24/16)	26/14	NA	68.00 ± 36.56d	CT				
8-Chen 2020 (24)	30 (13/17)	NA	62.00 ± 5.12	42.00 ± 5.52d	SA + CT	4 weeks	②③	NA	None
	30 (14/16)	NA	61.77 ± 4.81	43.40 ± 5.10d	CT				
9-Liu 2013 (25)	25 (10/15)	NA	NA	NA	EA + CT	4 weeks	①	NA	None
	25 (10/15)	NA	NA	NA	CT				
10-Chen 2020 (26)	48 (26/22)	NA	60.47 ± 2.35	42.66 ± 7.89d	SA + BA	4 weeks	①	NA	None
	48 (25/23)	NA	60.80 ± 2.19	42.50 ± 7.31d	CT				
11-Ding 2016 (27)	40 (24/16)	NA	56.24 ± 8.12	14.24 ± 9.58d	SA + CT	8 weeks	①③	NA	None
	46 (24/22)	NA	57.87 ± 9.01	11.84 ± 10.41d	CT				
12-Du 2019 (28)	30 (14/16)	NA	62.14 ± 9.48	26.00 ± 0.01d	SA + CT	8 weeks	①②	NA	None
	30 (15/15)	NA	66.49 ± 10.03	25.00 ± 0.01d	CT				
13-Duan 2021 (29)	41 (25/26)	NA	44.08 ± 5.24	3.65 ± 0.47 m	SA + BA	4 weeks	①	NA	None
	41 (23/18)	NA	43.30 ± 5.61	3.28 ± 0.19 m	CT				
14-Feng 2014 (30)	30 (16/14)	NA	64 ± 6	6.05 ± 1.29 m	WA + CT	8 weeks	②③	NA	None
	30 (21/9)	NA	72 ± 8	5.95 ± 1.42 m	CT				
15-Han 2021 (31)	30 (18//12)	22//8	54.43 ± 9.34	NA	SA + CT	8 weeks	①②	NA	None
	30 (16/14)	20//10	58.86 ± 10.47	NA	CT				
16-Hu 2019 (32)	40 (27/13)	NA	45.87 ± 10.22	28.2 ± 8.5d	WA + CT	4 weeks	②③	NA	None
	40 (24/16)	NA	46.59 ± 10.18	27.6 ± 9.1d	CT				
17-Niu 2021 (33)	75 (40/35)	NA	52.06 ± 7.98	NA	SA + CT	6 weeks	①②③	NA	None
	75 (41/34)	NA	51.89 ± 10.24	NA	CT				
18-Kong 2021 (34)	78 (40/35)	NA	52.06 ± 7.98	NA	SA + CT	6 weeks	①②③	NA	None
	78 (41/34)	NA	51.89 ± 10.24	NA	CT				
19-Leng 2020 (35)	40 (21/19)	18/22	60.65 ± 7.23	34.58 ± 8.21d	WA + CT	8 weeks	①③	NA	2/3
	40 (22/18)	16/24	61.35 ± 8.34	32.83 ± 8.35d	BA+CT				
20-Chen 2016 (36)	30 (12/18)	NA	NA	NA	SA + BA	8 weeks	①	NA	1/2
	30 (15/15)	NA	NA	NA	CT				
21-Li 2017 (37)	32 (17/15)	NA	59.15 ± 5.07	NA	WA + CT	4 weeks	②	NA	None
	30 (18//12)	NA	58.25 ± 5.47	NA	CT				
22-Lin 2020 (38)	34 (22/12)	NA	66.69 ± 6.28	NA	SA + BA	8 weeks	①	None	None
	34 (21/18)	NA	67.98 ± 7.40	NA	CT				
23-Lin 2014 (39)	30 (18/12)	NA	65 ± 5	29.85 ± 18.10d	EA + CT	4 weeks	②	NA	None
	29 (19/10)	NA	67 ± 7	30.05 ± 19.89d	CT				
24-Qian 2018 (40)	35 (17/18)	NA	70 ± 6	10.14 ± 3.37	SA + CT	8 weeks	①	NA	None
	35 (20/15)	NA	69 ± 6	10.54 ± 3.85	CT				
25-Feng 2015 (41)	30 (16/14)	14/16	NA	NA	AB+CT	4 weeks	①③	NA	None
	30 (19/11)	15/15	NA	NA	CT				
26-Song 2020 (42)	35 (19/16)	26//9	60 ± 10	2.8 ± 1.4 m	SA + BA	8 weeks	②③	NA	None
	35 (21/14)	25//10	58 ± 10	2.5 ± 1.6 m	CT				
27-Sun 2019 (43)	50 (22/28)	29/21	60.21 ± 2.12	73.50 ± 5.47d	SA + BA	4 weeks	①	YES	None
	50 (23/27)	28/22	60.28 ± 2.36	73.25 ± 5.11d	BA+CT				
28-Tan 2020 (44)	50 (25/25)	NA	58.9 ± 6.5	NA	SA + BA	8 weeks	①	NA	None
	50 (26/24)	NA	58.3 ± 6.3	NA	CT				
29-Ge 2016 (45)	50 (29/21)	NA	NA	NA	SA + BA	8 weeks	①	NA	None
	50 (30/20)	NA	NA	NA	CT				
30-Tian 2021 (46)	25 (14/11)	21//4	68.19 ± 0.16	NA	SA + BA	8 weeks	①	NA	None
	25 (15/10)	20//5	68.21 ± 0.15	NA	CT				
31-Wang 2015 (47)	40 (21/19)	31/9	66.3 ± 6.1	NA	AA+CT	4 weeks	①③	NA	None
	40 (20/20)	32/8	65.6 ± 7.6	NA	BA+CT				
32-Wang 2021 (48)	40 (26/14)	NA	66 ± 8	14.62 ± 6.17	SA + CT	8 weeks	①②③	NA	None
	40 (24/16)	NA	67 ± 9	15.45 ± 6.24	CT				
33-Wang 2021 (49)	30 (18/12)	16/14	51.07 ± 8.04	38.27 ± 8.14d	SA + CT	4 weeks	①②③	NA	None
	30 (17/13)	18//12	56.57 ± 9.13	39.07 ± 9.14d	CT				
34-Wang 2017 (50)	30 (20/10)	NA	53.27 ± 11.62	NA	EA + CT	8 weeks	①③	NA	None
	30 (19/11)	NA	56.73 ± 9.32	NA	CT				
35-Wang 2015 (51)	38 (24/14)	20/18	46.89 ± 6.10	2.40,2.11 m	WA + SA	4 weeks	②	NA	None
	38 (25/13)	21/17	44.44 ± 9.92	2.38,2.16 m	CT				
36-Wang 2018 (52)	50 (24/26)	27/23	53.8 ± 11.7	NA	SA + CT	3 weeks	①②	NA	None
	50 (26/24)	29/21	54.5 ± 13.6	NA	CT				
37-Wang 2011 (53)	20 (10/10)	NA	61.39 ± 10.42	30.05 ± 19.89d	SA + CT	4 weeks	①	NA	None
	20 (12/8)	NA	58.65 ± 9.53	31.70 ± 21.75d	SA				
	20 (13/7)	NA	62.29 ± 11.18	29.85 ± 18.10d	CT				
38-Lei 2021 (54)	34 (19/15)	21/13	59.73 ± 6.82	4.08 ± 0.72 m	SA + BA	8 weeks	②③	NA	None
	34 (18/16)	20/14	59.40 ± 6.74	4.11 ± 0.70 m	BA+CT				
39-Wang 2018 (55)	40 (23/17)	NA	45.39 ± 11.42	24.05 ± 11.89	SA + CT	4 weeks	①	NA	None
	40 (28/12)	NA	42.29 ± 11.72	26.85 ± 16.10	SA				
	40 (26/14)	NA	52.65 ± 7.53	19.70 ± 11.75	CT				
40-Wang 2019 (56)	104 (62/42)	NA	62 ± 7	54.39 ± 9.57	SA + BA	4 weeks	①	NA	None
	104 (61/43)	NA	62 ± 7	55 ± 10	CT				
41-Zhang 2021 (57)	50 (24/26)	NA	63.5 ± 3.4	NA	SA + BA	6 weeks	①	NA	None
	50 (25/25)	NA	60.32 ± 7.93	NA	CT				
42-Wei 2019 (58)	30 (18/12)	NA	60.32 ± 7.93	NA	EA + CT	6 weeks	②	NA	None
	30 (19/11)	NA	60.38 ± 8.01	NA	CT				
43-Zhao 2021 (59)	31 (17/14)	NA	53.61 ± 5.69	3.75 ± 1.46d	SA + AB	8 weeks	①	NA	None
	31 (20/11)	NA	54.23 ± 6.27	3.89 ± 1.50d	CT				
44-Xu 2022 (60)	45 (28/17)	30/15	67.85 ± 1.91	1.94 ± 0.34 m	BA+CT	8 weeks	①③	NA	None
	45 (30/15)	32/13	68.02 ± 2.03	2.05 ± 0.37 m	CT				
45-Zheng 2021 (61)	44 (26/18)	12/32	62 ± 6	54.7 ± 14.2d	SA + BA	8 weeks	②③	NA	None
	43 (32/11)	13/30	61 ± 7	56.9 ± 16.4d	BA+CT				
46-Yan 2022 (62)	30 (15/15)	24//6	67.4 ± 4.2	32.2 ± 9.01d	WA + CT	4 weeks	①②	NA	None
	30 (17/13)	20//10	67.2 ± 4.9	31.3 ± 10.3d	CT				
47-Yang 2018 (63)	34 (18/16)	NA	68 ± 8 7	14.7 ± 6.2	SA + CT	4 weeks	①②	NA	None
	33 (19/14)	NA	67 ± 8	15.5 ± 6.2	CT				
48-Yang 2020 (64)	17 (11/6)	10//7	65.5 ± 6.8	20.5 ± 3.0	SA + BA	4 weeks	①	NA	None
	17 (10/7)	8//9	65.8 ± 5.6	20.0 ± 3.5	CT				
49-Zhou 2021 (65)	52 (29/23)	18/34	58.17 ± 6.64	25.43 ± 3.17d	SA + BA	2 weeks	①	NA	None
	52 (27/25)	17/35	57.63 ± 7.02	25.34 ± 2.98d	BA				
50-Yang 2019 (66)	40 (24/16)	NA	51.35 ± 7.30	21.40 ± 5.38d	SA + BA	4 weeks	①	NA	None
	40 (22/18)	NA	51.72 ± 7.46	21.57 ± 5.54d	CT				
51-Yao 2019 (67)	40 (21/19)	NA	61.27 ± 5.38	42.38 ± 14.23d	EA + CT	12 weeks	②	YES	None
	38 (20/18)	NA	62.72 ± 6.48	44.23 ± 18.87d	CT				
52-Yao 2020 (68)	30 (11/19)	21//9	54.6 ± 11.8	2.5 ± 0.8 m	SA + BA	4 weeks	①②	NA	None
	30 (9/21)	18//12	57.4 ± 12.8	2.4 ± 1.0 m	CT				
53-Yu 2021 (69)	30 (18/12)	NA	59 ± 3	NA	AA+CT	4 weeks	②	NA	None
	30 (17/13)	NA	59 ± 3	NA	CT				
54-Zhan 2016 (70)	25 (14/11)	15//10	60 ± 10	78.2 ± 47.2	SA + CT	4 weeks	①②	NA	None
	25 (19/6)	13//12	60 ± 9	75.8 ± 50.2	BA+CT				
55-Zhang 2020 (71)	30 (20/10)	NA	70. 10 ± 4. 51	23. 03 ± 7. 47d	EA + CT	6 weeks	②	NA	None
	30 (18/12)	NA	69. 03 ± 4. 70	24. 63 ± 11. 77d	CT				
56-Zhang 2018 (72)	65 (45/20)	52/13	59.87 ± 9.78	77.9 ± 21.85	SA + BA	4 weeks	①②	NA	None
	65 (47/18)	50/15	59.57 ± 8.85	75.5 ± 19.16	BA				
57-Zhang 2019 (73)	35 (20/15)	25//10	59.95 ± 8.71	39.72 ± 18.73d	AA+BA	4 weeks	①③	NA	None
	35 (19/16)	16/19	61.12 ± 9.62	42.11 ± 17.56d	CT				
58-Zheng 2019 (74)	29 (18/11)	NA	63 ± 3	31.78 ± 16.15d	WA + CT	12 weeks	②③	NA	None
	28 (19/9)	NA	67 ± 7	29.85 ± 18.36d	CT				
59-Zhou 2022 (75)	75 (41/34)	NA	50.92 ± 11.16	4.01 ± 0.61d	OA + CT	6 weeks	①	NA	None
	75 (41/34)	NA	51.01 ± 13.19	3.97 ± 0.56d	CT				
60-Zhuo 2021 (76)	20 (12/8)	NA	63.25 ± 9.34	2.15 ± 1.03 m	SA + BA	3 weeks	②	NA	None
	22 (12/10)	NA	63.04 ± 9.16	2.27 ± 1.06 m	CT				
61-Zhou 2020 (77)	30 (18/12)	NA	53.76 ± 9.27	64.35 ± 31.65d	SA + BA	8 weeks	①②	NA	None
	30 (20/10)	NA	53.89 ± 9.52	64.15 ± 30.97d	CT				
62-Zhu 2014 (78)	40 (22/18)	18/22	55.56 ± 13.58	21.67 ± 15.82d	SA	4 weeks	①	NA	None
	40 (24/16)	21/19	56.37 ± 13.26	20.51 ± 13.38d	BA				

T, Treatment group; C, Control group; M, Man; F, Female; I, Ischemic Stroke; H, Hemorrhagic stroke; SA, Scale acupuncture; BA, Body acupuncture; OA, Ophthalmic acupuncture; WA, warm acupuncture; EA, Electro-acupuncture; AB, Auricular bloodletting; AA, Abdominal acupuncture; CT, Cognitive training; ①, Mental State Examination Scale (MMSE); ②, Montreal Cognitive Assessment Scale (MoCA); ③, Modified Barthel Index scale (MBI) NA, Not mentioned.

### 3.3. Risk of bias

According to ROB2, three studies (23, 35, 36) were rated at high risk of bias, two studies (19, 26) were rated at low risk of bias, and the remaining studies were rated at some risk of bias. The main issues included lack of description of allocation concealment, lack of blinding of outcome assessors, and lack of exploration of prospective protocols, which ultimately led to an increased risk of bias. The risk of bias assessment for the included studies is presented in Supplementary Figure S1. Many of the items in the CONSORT (18/25 items) statements did not achieve the desired reporting rate (>80%), which was shown in Supplementary Appendix 3.

### 3.4. Pairwise meta-analysis

After a comprehensive analysis of studies with the same treatment and outcomes, we conducted 19 direct-paired meta-analyzes to compare MMSE scores, 11 to compare MoCA scores, and 10 to compare MBI scores, respectively. As for MMSE scores，SA (two RCTs; WMD = 2.50, 95%CI: 0.67, 4.32, *p* = 0.007), BA+CT (one RCT; WMD = 5.29, 95%CI: 4.16, 6.42, P<0.00001), SA + CT (16 RCTs; WMD = 4.39, 95%CI: 3.08, 5.70, P<0.00001), OA + CT (one RCT; WMD = 5.85, 95%CI: 4.69, 7.01, P<0.00001), WA + CT (one RCT; WMD = 1.86, 95%CI: 0.16, 3.56, *p* = 0.03), AB+CT (one RCT; WMD = 2.57, 95%CI: 0.81, 4.33, *p* = 0.004), SA + BA (16 RCTs; WMD = 4.24, 95%CI: 3.28, 5.20, *p*<0.00001), SA + AB (one RCT; WMD = 3.84, 95%CI: 2.00, 5.68, *p*<0.00001) were effective than the CT group. In the comparison of different acupuncture treatments, we found that SA (one RCT; WMD = 0.88, 95%CI: 0.15, 1.61, *p* = 0.02), SA + CT (one RCT; WMD = 2.42, 95%CI: 0.74, 4.10, *p* = 0.005), SA + BA (two RCTs; WMD = 1.79. 95% CI: 1.05, 2.53, *p* < 0.00001) improved MMSE scores more than BA alone. Furthermore, SA + CT (one RCT; WMD = 2.48, 95%CI: 0.18, 4.78, *p* = 0.03) and WA + CT (one RCT; WMD = 1.22, 95%CI: 0.08, 2.36, *p* = 0.04) were more effective in improving MMSE scores compared with BA+CT. Meanwhile, SA + CT had a greater effect than SA alone (two RCTs; WMD = 3.23, 95%CI: 1.49, 4.97, *p* = 0.0003). However, there was no statistical difference in efficacy between BA and CT, EA and CT, AA+BA and CT, AA+CT and BA+CT, and SA + AB and BA+CT. In terms of MoCA scores, SA + CT (10 RCTs; WMD =3.75, 95%CI: 2.81, 4.69, P<0.00001), SA + BA (four RCTs; WMD = 3.26, 95%CI: 2.19, 4.34, P<0.00001), WA + CT (five RCTs; WMD = 3.96, 95%CI: 2.29, 5.62, P<0.00001), EA + CT (four RCTs; WMD = 3.16, 95%CI: 1.88, 4.45, *p*<0.00001), AA+CT (one CRT; WMD = 3.00, 95%CI: 0.26, 5.74, *p* = 0.03) were more effective than CT alone. Furthermore, compared with WA + CT, SA + CT (one RCT; WMD = 3.14, 95%CI: 2.15, 4.13, *p*<0.00001) and SA + BA (two RCTs; WMD = 3.61, 95%CI: 2.11, 5.11, *p*<0.00001) were more effective in improving MoCA scores. Meanwhile, SA + BA had a greater effect than BA alone (one RCT; WMD = 2.00, 95%CI: 1.37, 2.63, *p*<0.00001). However, there was no statistical difference in efficacy between SA + CT and SA, SA and CT, and WA + SA and CT. For MBI scores, BA+CT (one RCT; WMD = 7.23, 95%CI: 5.39, 9.07, *p*<0.00001), SA + CT (seven RCTs; WMD = 12.83, 95%CI: 5.06, 20.60, *p* = 0.001), WA + CT (three RCTs; WMD = 10.13, 95%CI: 5.31, 14.96, *p*<0.0001), SA + BA (two RCTs; WMD = 19.13, 95%CI: 18.08, 20.18, *p*<0.00001), EA + CT (one RCT; WMD = 1.74, 95%CI: 0.13, 3.35, *p* = 0.03) and AB + CT (one RCT; WMD =13.57, 95%CI: 6.23, 20.82, *p* = 0.0002) were more effective than CT group. Furthermore, compared with BA+CT, AA+CT (one RCT; WMD =6.00, 95%CI: 6.40, 7.40, *p*<0.00001), SA + BA (two RCTs; WMD = 10.40, 95%CI: 3.14, 17.66, *p* = 0.005) and WA + CT (one RCT; WMD = 3.23, 95%CI: 0.12, 6.34, *p* = 0.04) were more effective in improving MBI scores. However, there was no statistical difference in efficacy between AA+BA and CT. The results of paired meta-analysis and heterogeneity are presented in Table 3.

**Table 3:** The results of the paired meta-analysis.

Comparison	WMD (95% CI)	Number of studies	Number of patients	I^2^ (%)	*p*-value
MMSE					
K-A	**4.24 [3.28, 5.20]**	16	1,481	94	<0.00001
E-A	**4.39 [3.08, 5.70]**	16	1,310	91	<0.00001
D-A	**5.29 [4.16, 6.42]**	1	90	–	–
F-A	**5.85 [4.69, 7.01]**	1	150	–	–
G-A	**1.86 [0.16, 3.56]**	1	60	–	–
I-A	**2.57 [0.81, 4.33]**	1	60	–	–
N-A	**3.84 [2.00, 5.68]**	1	62	–	–
E-B	**2.42 [0.74, 4.10]**	1	104	–	–
E-C	**3.23 [1.49, 4.97]**	2	120	0	0.68
E-D	**2.48 [0.18, 4.78]**	1	50	–	–
G-D	**1.22 [0.08, 2.36]**	1	75	–	–
K-B	**1.79 [1.05, 2.53]**	2	234	17	0.27
C-A	**2.50 [0.67, 4.32]**	2	120	0	1
C-B	**0.88 [0.15, 1.61]**	1	80	–	–
B-A	0.04 [−1.63, 1.71]	1	103	–	–
H-A	4.07 [−0.45, 8.60]	2	110	96	<0.00001
L-A	2.57 [−0.14, 5.28]	1	58	–	–
J-D	0.90 [−0.20, 2.00]	1	80	–	–
K-D	0.94 [−0.18, 2.06]	1	100	–	–
MoCA					
E-A	**3.75 [2.81, 4.69]**	10	901	79	<0.00001
K-A	**3.26 [2.19, 4.34]**	4	505	0	0.71
G-A	**3.96 [2.29, 5.62]**	5	419	88	<0.00001
H-A	**3.16 [1.88, 4.45]**	4	310	0	0.92
J-A	**3.00 [0.26, 5.74]**	1	50	–	–
E-D	**3.14 [2.15, 4.13]**	1	60	–	–
K-D	**3.61 [2.11, 5.11]**	2	128	67	0.08
K-B	**2.00 [1.37, 2.63]**	1	57	–	–
E-C	2.08 [−0.32, 4.48]	1	104	–	–
C-A	−0.06 [−2.34, 2.22]	1	103	–	–
M-A	1.47 [−0.87, 3.81]	1	76	–	–
MBI					
D-A	**7.23 [5.39, 9.07]**	1	90	–	–
E-A	**12.83 [5.06, 20.60]**	7	647	97	<0.00001
G-A	**10.13 [5.31, 14.96]**	3	197	64	0.06
K-A	**19.13 [18.08, 20.18]**	2	156	46	0.17
H-A	**1.74 [0.13, 3.35]**	1	60	–	–
I-A	**13.57 [6.32, 20.82]**	1	60	–	–
J-D	**6.00 [4.60, 7.40]**	1	80	–	–
K-D	**10.40 [3.14, 17.66]**	2	155	74	0.05
G-D	**3.23 [0.12, 6.34]**	1	75	–	–
L-A	2.00 [−2.95, 6.95]	1	58	–	–

The bold font indicates a statistical difference; A, Cognitive training; B, Body acupuncture; C, Scalp acupuncture; D, Body acupuncture plus cognitive training; E, Scalp acupuncture plus cognitive training; F, Ophthalmic acupuncture plus cognitive training; G, Warm acupuncture plus cognitive training; H, Electro-acupuncture plus cognitive training; I, Auricular bloodletting plus cognitive training; J, Abdominal acupuncture plus cognitive training; K, Scalp acupuncture plus body acupuncture; L, Abdominal acupuncture plus body acupuncture; M, Warm acupuncture plus scalp acupuncture; N, Scalp acupuncture plus auricular bloodletting.

### 3.5. Network meta-analysis

**Figure 2:**
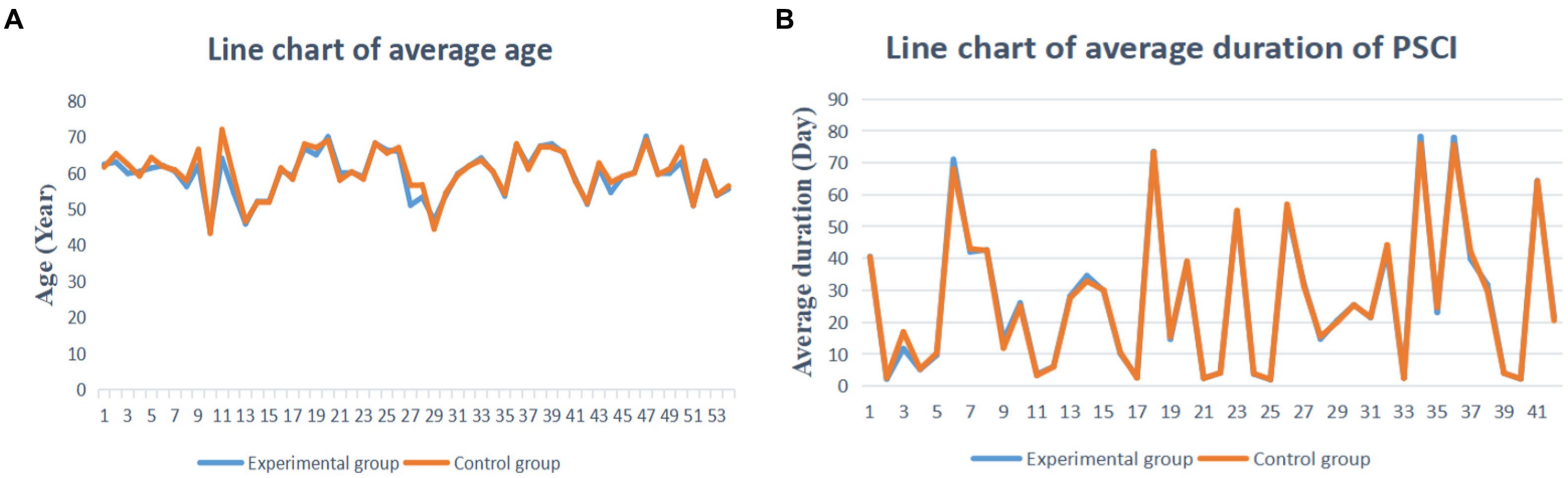
Baseline assessment. **(A)** The average age assessment. **(B)** The average duration PSCI assessment.

The transferability hypothesis was assessed by evaluating the baseline differences in mean age and disease duration of patients in the included studies. As shown in Figure 2A, the mean age of PSCI patients showed a high degree of similarity among the included studies. Furthermore, as shown in Figure 2B, the mean duration of disease in patients with PSCI also showed a high degree of similarity. Therefore, this study satisfies the transferability hypothesis and reliable results can be obtained.

The inconsistency test results for MMSE, MoCA, and MBI scores were all greater than 0.05 (*p* = 0.9064, 0.7492, and 0.9231), so the consistency model was selected for analysis. We adopted the node-splitting method to test the internal inconsistency of NMA. The results showed no significant differences between direct or indirect comparisons for each split node (*p* > 0.05), which suggests no evidence of the existence of inconsistency (Supplementary Figure S2). The results of the closed-loop inconsistency test showed that all 95% CI contained 0, which indicated that the closed-loop comparisons possessed excellent consistency (Supplementary Table S1). The Brooks-Gelman-Rubin diagnostic plots showed that the shrink factor’s median and 97.5% value tended to be 1 and stabilized after 5,000 iterations, and then the Bayesian model was calculated up to 20,000 iterations (Supplementary Figure S3). Meanwhile, we observed the trajectory and density plots (Supplementary Figure S4). All these results indicate that the model has excellent convergence.

**Figure 3:**
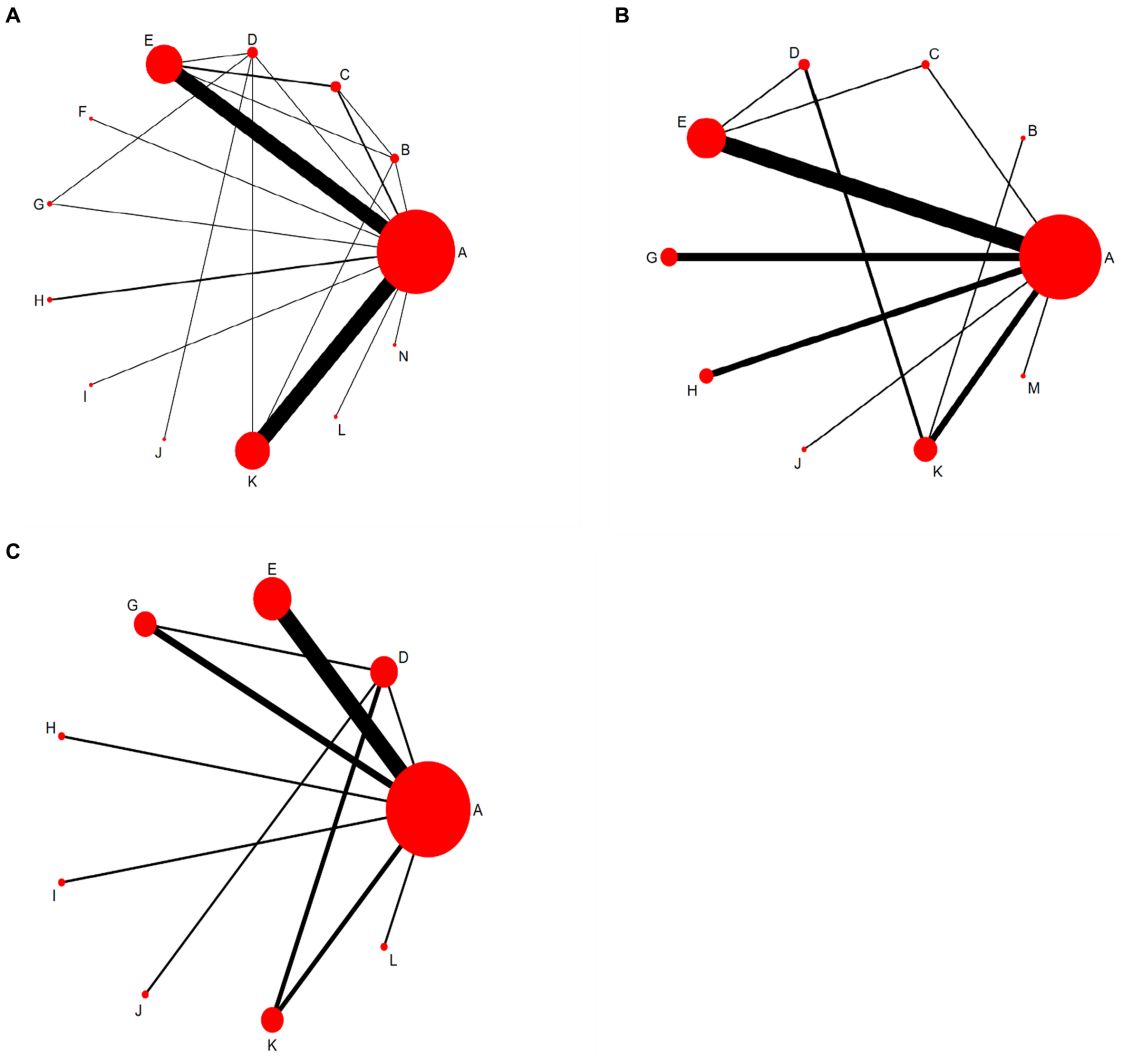
Network evidence diagram. A, Cognitive training; B, Body acupuncture; C, Scalp acupuncture; D, Body acupuncture plus cognitive training; E, Scalp acupuncture plus cognitive training; F, Ophthalmic acupuncture plus cognitive training; G, Warm acupuncture plus cognitive training; H, Electro-acupuncture plus cognitive training; I: Auricular bloodletting plus cognitive training; J, Abdominal acupuncture plus cognitive training; K, Scalp acupuncture plus body acupuncture; L: Abdominal acupuncture plus body acupuncture; M: Warm acupuncture plus scalp acupuncture; N: Scalp acupuncture plus auricular bloodletting. **(A)** The Minimum Mental State Examination scale (MMSE). **(B)** The Montreal Cognitive Assessment Scale (MoCA). **(C)** The Modified Barthel Index scale (MBI).

All included studies with 4,057 participants and 13 acupuncture-related therapies reported MMSE data (Figure 3A), including 3 (18, 53, 55) three-arm studies (6.4%) and 44 two-arm studies (93.6%). Among them, the CT group had the largest sample size. The two groups most commonly compared were scalp acupuncture plus cognitive training and cognitive training, and scalp acupuncture plus body acupuncture and cognitive training, respectively. 29 studies reported MoCA data involving 2,285 participants and 10 acupuncture-related therapies (Figure 3B). 20 studies reported MBI data involving 1,579 participants and 9 acupuncture-related therapies (Figure 3C).

In terms of improving MMSE scores, the results (Table 4) showed that BA+CT (MD = −3.00, 95%CI: −5.94, −0.05), SA + CT (MD = −4.50, 95%: −5.85, −3.16), OA + CT (MD = −6.31, 95%CI: −11.64, −1.01) and SA + BA (MD = −3.92, 95%CI: −5.19, −2.64) were more effective for PSCI patients compared to the CT group. In addition, SA + CT (MD = −2.94, 95%CI: −5.85, −0.04) was more effective than BA when comparing different acupuncture treatments. Regarding the improvement of MoCA score, the results (Table 4) showed that SA + CT (MD = 3.54, 95%CI: 2.44, 4.59), WA + CT (MD = 4.04, 95%CI: 2.53, 5.50), EA + CT (MD = 3.33, 95%CI: 1.29, 5.38) and SA + BA (MD = 2.84, 95%CI: 1.10, 4.56) were more effective in patients with PSCI compared to the CT group. In addition, SA + CT (MD = 3.71, 95%CI: 1.43, 5.98), WA + CT (MD = 4.20, 95%CI: 1.51, 6.88), EA + CT (MD = 3.49, 95%CI: 0.45, 6.54) and SA + BA (MD = 3.01, 95%CI: 1.00, 4.96) were more effective than BA+CT when comparing different acupuncture treatments. In terms of improving MBI scores, the results (Table 5) showed that SA + CT (MD = −12.80, 95%CI: −17.93, −7.67) and EA + CT (MD = −10.59, 95%CI: −19.05, −2.14) were more effective than CT alone.

**Table 4:** Network meta-analysis results of Minimum Mental State Examination Scale (MMSE) and Montreal Cognitive Assessment Scale (MoCA).

Treatment	MoCA													
MMSE	**A**	0.87 [−2.71, 4.40]	0.68 [−2.72, 4.11]	−0.16 [−2.41, 2.06]	**3.54 [2.44, 4.59]**	–	**4.04 [2.53, 5.50]**	**3.33 [1.29, 5.38]**	–	3.33 [−0.68, 7.36]	2.84 [1.10, 4.56]	–	1.40 [−2.41, 5.25]	–
	−1.55 [−4.29, 1.19]	**B**	−0.17 [−5.04, 4.75]	−1.02 [−4.69, 2.71]	2.68 [−0.96, 6.63]	–	3.17 [−0.65, 7.07]	2.46 [−1.64, 6.59]	–	2.47 [−2.89, 7.84]	1.97 [−1.11, 5.11]	–	0.54 [−4.67, 5.82]	–
	−2.21 [−5.31, 0.88]	−0.66 [−4.12, 2.81]	**C**	−0.85 [−4.88, 3.18]	2.85 [−0.59, 6.27]	–	3.35 [−0.41, 7.08]	2.65 [−1.37, 6.65]	–	2.64 [−2.62, 7.90]	2.16 [−1.65, 5.95]	–	0.72 [−4.39, 5.82]	–
	**−3.00 [−5.94, −0.05]**	−1.45 [−5.38, 2.50]	−0.78 [−4.98, 3.41]	**D**	**3.71 [1.43, 5.98]**	–	**4.20 [1.51, 6.88]**	**3.49 [0.45, 6.54]**	–	3.49 [−1.11, 8.09]	**3.01 [1.00, 4.96]**	–	1.57 [−2.84, 6.02]	–
	**−4.50 [−5.85, −3.16]**	**−2.94 [−5.85, −0.04]**	−2.27 [−5.42, 0.84]	−1.49 [−4.57, 1.55]	**E**	–	0.49 [−1.32, 2.31]	−0.21 [−2.50, 2.08]	–	−0.20 [−4.36, 3.91]	−0.70 [−2.63, 1.23]	–	−2.14 [−6.08, 1.86]	–
	**−6.31 [−11.64, −1.01]**	−4.73 [−10.75, 1.18]	−4.09 [−10.21, 2.09]	−3.30 [−9.36, 2.73]	−1.80 [−7.26, 3.68]	**F**	–	–	–	–	–	–	–	–
	−3.32 [−7.37, 0.75]	−1.76 [−6.66, 3.11]	−1.10 [−6.20, 3.97]	−0.31 [−4.38, 3.69]	1.18 [−3.02, 5.40]	2.98 [−3.68, 9.67]	**G**	−0.70 [−3.22, 1.83]	–	−0.70 [−4.93, 3.61]	−1.19 [−3.46, 1.08]	–	−2.62 [−6.71, 1.49]	–
	−3.25 [−6.99, 0.51]	−1.69 [−6.35, 2.96]	−1.03 [−5.89, 3.84]	−0.26 [−5.00, 4.55]	1.24 [−2.72, 5.26]	3.04 [−3.40, 9.60]	0.06 [−5.45, 5.62]	**H**	–	0.03 [−4.51, 4.51]	−0.48 [−3.16, 2.18]	–	−1.91 [−6.26, 2.39]	–
	−2.66 [−8.15, 2.76]	−1.10 [−7.24, 4.98]	−0.45 [−6.75, 5.83]	0.33 [−5.91, 6.51]	1.83 [−3.83, 7.43]	3.63 [−4.00, 11.20]	0.64 [−6.15, 7.45]	0.58 [−6.10, 7.15]	**I**	–	–	–	–	–
	−4.90 [−10.97, 1.13]	−3.34 [−9.91, 3.21]	−2.69 [−9.46, 4.06]	−1.90 [3.98, 2.10]	−0.40 [−6.51, 5.70]	1.40 [−6.64, 9.48]	−1.58 [−8.12, 5.07]	−1.63 [−8.82, 5.45]	−2.24 [−10.42, 5.90]	**J**	−0.49 [−4.86, 3.86]	–	−1.93 [−7.44, 3.65]	-
	**−3.92 [−5.19, −2.64]**	−2.36 [5.11, 0.37]	−1.70 [−4.97, 1.55]	−0.91 [−3.98, 2.10]	0.57 [−1.20, 2.39]	2.37 [−3.05, 7.86]	−0.60 [−4.80, 3.59]	−0.67 [−4.63, 3.28]	−1.25 [−6.84, 4.40]	0.98 [−5.09, 7.07]	**K**	–	−1.43 [−5.63, 2.78]	-
	−3.14 [−8.85, 2.53]	−1.59 [−7.84, 4.72]	−0.93 [−7.31, 5.61]	−0.11 [−6.52, 6.29]	1.36 [−4.46, 7.20]	3.18 [−4.56, 10.97]	0.18 [−6.79, 7.17]	0.11 [−6.69, 6.96]	−0.47 [−8.37, 7.46]	1.76 [−6.53, 10.07]	0.77 [−5.06, 6.62]	**L**	–	–
	–	–	–	–	–	–	–	–	–	–	–	–	**M**	–
	−3.41 [−8.83, 2.02]	−1.85 [−7.98, 4.24]	−1.19 [−7.44, 5.03]	−0.39 [−6.60, 5.73]	1.08 [−4.50, 6.71]	2.89 [−4.69, 10.42]	−0.07 [−6.93, 6.67]	−0.14 [−6.79, 6.38]	−0.73 [−8.40, 6.96]	1.49 [−6.61, 9.60]	0.50 [−5.08, 6.09]	−0.27 [−8.14, 7.58]	-	**N**

The bold font indicates a statistical difference; A, Cognitive training; B, Body acupuncture; C, Scalp acupuncture; D, Body acupuncture plus cognitive training; E, Scalp acupuncture plus cognitive training; F, Ophthalmic acupuncture plus cognitive training; G, Warm acupuncture plus cognitive training; H, Electro-acupuncture plus cognitive training; I, Auricular bloodletting plus cognitive training; J, Abdominal acupuncture plus cognitive training; K, Scalp acupuncture plus body acupuncture; L, Abdominal acupuncture plus body acupuncture; M, Warm acupuncture plus scalp acupuncture; N, Scalp acupuncture plus auricular bloodletting.

**Table 5:** Network meta-analysis results of Modified Barthel Index scale (MBI).

Treatment	**A**								
MBI	−7.31 [−20.42, 5.81]	**D**							
	**−12.80 [−17.93, −7.67]**	−5.49 [−19.57, 8.59]	**E**						
	−1.58 [−22.03, 18.88]	−3.29 [−18.89, 12.32]	2.20 [−7.69, 12.09]	**G**					
	**−10.59 [−19.05, −2.14]**	6.91 [−11.56, 25.38]	12.40 [−1.58, 26.38]	10.19 [−5.23, 25.71]	**H**				
	−0.40 [−13.14, 12.61]	−7.01 [−26.49, 12.47]	−1.52 [−16.81, 13.77]	−3.73 [−20.43, 13.98]	−13.92 [−33.33, 5.49]	**I**			
	−14.23 [−28.72, 0.08]	−4.98 [−17.97, 8.00]	0.51 [−18.63, 19.64]	−1.70 [−21.98, 18.58]	−11.89 [−34.45, 10.67]	2.03 [−21.36, 25.42]	**J**		
	−12.29 [−30.72, 6.14]	−7.77 [−24.38, 8.83]	−2.28 [−13.69, 9.12]	−4.49 [−17.72, 8.74]	−14.68 [−31.21, 1.84]	−0.76 [−18.40, 16.88]	−2.79 [−23.85, 18.27]	**K**	
	−3.93 [−23.21, 15.35]	6.31 [−12.75, 25.36]	11.80 [−2.95, 26.54]	9.59 [−6.61, 25.80]	−0.60 [−19.58, 18.38]	13.32 [−6.64, 33.28]	11.29 [−11.75, 34.33]	14.08 [−3.09, 31.26]	**L**

The bold font indicates a statistical difference; A, Cognitive training; D, Body acupuncture plus cognitive training; E, Scalp acupuncture plus cognitive training; G, Warm acupuncture plus cognitive training; H, Electro-acupuncture plus cognitive training; I, Auricular bloodletting plus cognitive training; J, Abdominal acupuncture plus cognitive training; K, Scalp acupuncture plus body acupuncture; L, Abdominal acupuncture plus body acupuncture.

**Figure 4:**
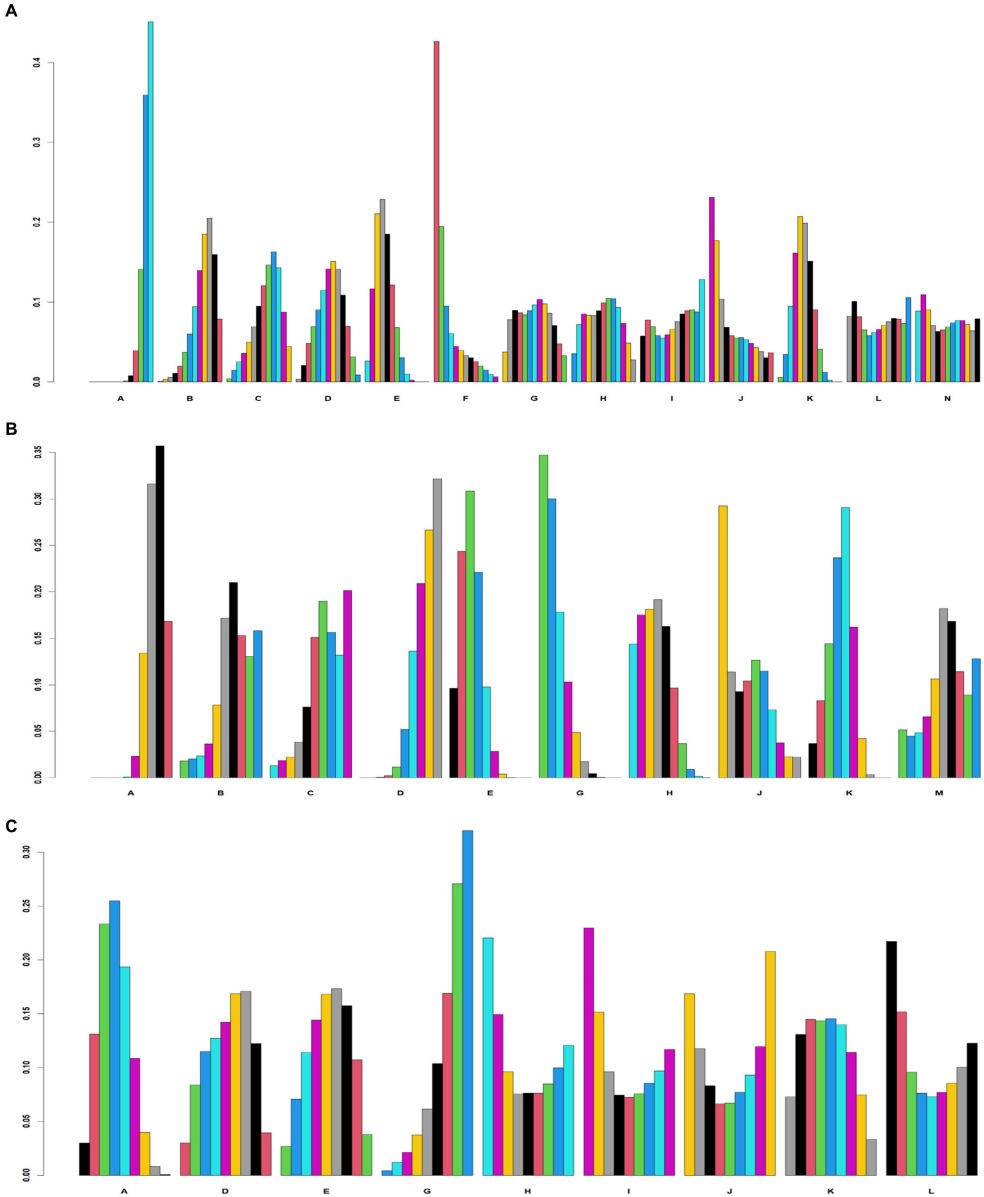
Probability ranking diagram. A, Cognitive training; B, Body acupuncture; C, Scalp acupuncture; D, Body acupuncture plus cognitive training; E, Scalp acupuncture plus cognitive training; F, Ophthalmic acupuncture plus cognitive training; G, Warm acupuncture plus cognitive training; H, Electro-acupuncture plus cognitive training; I, Auricular bloodletting plus cognitive training; J, Abdominal acupuncture plus cognitive training; K, Scalp acupuncture plus body acupuncture; L, Abdominal acupuncture plus body acupuncture; M, Warm acupuncture plus scalp acupuncture N: Scalp acupuncture plus auricular bloodletting. **(A)** Minimum Mental State Examination scale (MMSE). **(B)** Montreal Cognitive Assessment Scale (MoCA). **(C)** Modified Barthel Index scale (MBI).

We calculated the SUCRA values for each intervention for probability ranking (Supplementary Table S2) and constructed probability ranking histograms using R software (Figure 4). Figure 4A shows that among the 13 treatments, CT (6.2%) had the worst ability to improve MMSE scores. In addition, the top three acupuncture treatments that improved MMSE scores were OA + CT (79.7%), SA + CT (73.7%), and AA+CT (69.5%). WA + CT (86.5%), SA + CT (77.3%), and EA + CT (72.1%) were the three best acupuncture treatments for improving MoCA scores (Figure 4B). Furthermore, SA + BA (87.5%), AB+CT (75.4%), and SA + CT (72.6%) were the three most effective acupuncture treatments among the nine treatments for improving MBI scores (Figure 4C).

### 3.6. Adverse effect

Of the 62 studies included, six studies (17, 18, 38, 43, 61, 67) (9%) reported adverse reactions (Supplementary Table S3). Three studies (17, 18, 38) reported no adverse reactions during treatment. One study (67) reported that patients in the electroacupuncture group experienced subcutaneous hematomas after treatment, but they recovered spontaneously without systematic treatment. Two studies (43, 61) reported that patients experienced adverse effects such as dizziness, pallor, and sleepiness, which were considered to be possibly related to the first time they received acupuncture treatment. In conclusion, acupuncture treatment seems to be safe and reliable, but there is no sufficient evidence to prove it.

### 3.7. Publication bias

**Figure 5:**
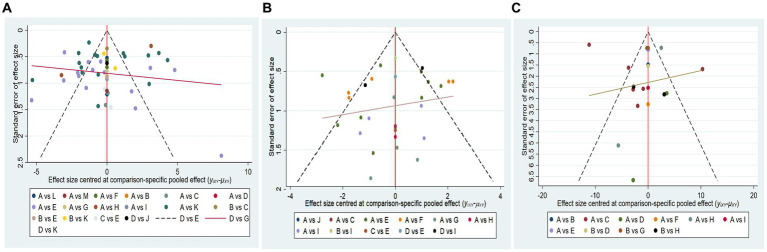
Comparative adjustment funnel plots. A, Cognitive training; B, Body acupuncture; C, Scalp acupuncture; D, Body acupuncture plus cognitive training; E, Scalp acupuncture plus cognitive training; F, Ophthalmic acupuncture plus cognitive training; G, Warm acupuncture plus cognitive training; H, Electro-acupuncture plus cognitive training; I, Auricular bloodletting plus cognitive training; J, Abdominal acupuncture plus cognitive training; K, Scalp acupuncture plus body acupuncture; L, Abdominal acupuncture plus body acupuncture; M, Warm acupuncture plus scalp acupuncture; N, Scalp acupuncture plus auricular bloodletting. **(A)** Minimum Mental State Examination scale (MMSE). **(B)** Montreal Cognitive Assessment Scale (MoCA). **(C)** Modified Barthel Index scale (MBI).

Comparative adjustment funnel plots were plotted using STATA software to assess publication bias and small sample size effects for the MMSE, MoCA, and MBI, respectively (Figure 5). The results showed that the comparative adjustment funnel plots for the MoCA and MBI scales were symmetrical, with most points evenly distributed on either side of the midline, reflecting a moderate sample size of the included studies and a low likelihood of publication bias. However, in the comparative adjusted funnel plot for the MMSE scale, most studies were more dispersed, with some of them lying outside the 95% CI range, indicating possible publication bias.

### 3.8. Evidence assessment of outcome measures

According to the GRADE scores, the strength of evidence for the three scales mentioned above ranged between very low and moderate. The main reasons for the reduced quality of evidence were flaws in study design and considerable statistical heterogeneity (Supplementary Table S4).

## 4. Discussion

### 4.1. Summary of main finding

In this systematic review, a total of 62 RCTs involving 5,073 participants and 13 acupuncture-related therapies were included. The results of the paired meta-analysis showed that most acupuncture-related therapies were effective in improving global cognitive ability (measured by MMSE and MoCA) and self-care of daily living (measured by MBI) in PSCI patients. Furthermore, BA, AA+BA, and WA + SA did not show sufficient advantages compared with the CT group. Notably, only one study of these three types of acupuncture therapy was compared with the CT group. The effect size may change as the sample size increases. The results of the NMA showed that in terms of improving MMSE scores, BA+CT, SA + CT, OA + CT, and SA + BA resulted in better outcomes for patients with PSCI compared with the CT group. In addition, the efficacy of SA + CT was better than BA alone. Based on the probability ranking results, it is clear that OA + CT is the most effective in improving MMSE scores. In terms of improving MoCA scores, SA + CT, WA + CT, EA + CT, and SA + BA were capable of delivering better outcomes than the CT group. Furthermore, the efficacy of the four acupuncture-related therapies was better than BA+CT. Based on the probability ranking results, it is clear that WA + CT is the most effective acupuncture therapy for improving MoCA scores. Regarding the improvement of MBI scores, SA + CT and EA + CT were more effective than CT alone. Furthermore, no statistically significant differences were found in the comparison of the various acupuncture therapies. Based on the probability ranking, it is clear that SA + BA is the most effective acupuncture measure for improving MBI scores in PSCI patients. Although the certainty of the evidence was rated as very low to moderate due to deficiencies in methodological quality and strong heterogeneity among studies, this review provides an up-to-date overview of the available RCTs of different types of acupuncture for PSCI.

### 4.2. Clinical practice applicability

Different from Alzheimer’s disease, there are still no symptomatic medications approved by authoritative official regulatory authorities for the treatment of PSCI (79). As a result, the focus of many medical associations has gradually shifted to whether nonpharmacologic interventions can help patients with PSCI improve cognitive function and maintain normal daily living independence (80). Based on clinical evidence, acupuncture is a relatively resource-intensive intervention that appears to be desirable for chronic conditions such as cognitive impairment that require long-term treatment (81). Studies (12, 13) have been conducted to demonstrate the effectiveness of acupuncture in the treatment of PSCI. However, acupuncture therapy encompasses a variety of forms and previous studies have tended to view acupuncture therapy as a whole. As for which acupuncture therapy can bring the best outcome for PSCI patients, it is still not clear. In clinical applications, ineffective acupuncture treatment inevitably delays optimal treatment time and wastes medical resources. Therefore, we conducted this study in an attempt to identify the best acupuncture treatment to improve cognitive function and self-care in daily life for patients with PSCI, providing new insights for clinical practice. We initially proposed pharmacological treatment and acupuncture techniques alone as the treatment measures for the control group. Unfortunately, we found that cholinesterase inhibitors are only used in China for the treatment of PSCI. Therefore, cognitive training and the acupuncture technique alone were finally set as the treatment measures for the control group in this study. The comparison between acupuncture therapy and cognitive training is meaningful because most guidelines on the treatment of PSCI include cognitive training as a treatment to improve PSCI patients.

The potential mechanisms of acupuncture for the treatment of PSCI have now been extensively studied. Therefore, we categorized the mechanisms into the following six aspects: (1) acupuncture can reduce the expression of inflammatory factors by inhibiting the degradation of IkB kinase or inhibiting the entry of NF-kB into the cell nucleus, which can result in the improvement of cognitive function (82), (2) acupuncture antagonizes neuronal apoptosis by increasing the transactivation activity of the PI3K/AKL signaling pathway (83), (3) acupuncture ameliorates neurological deficits by inhibiting the expression of autophagy-related proteins LC3-II and Beclin-1 in brain tissue (84), (4) acupuncture promotes axonal regeneration and improves synaptic plasticity by promoting the expression of PSD-95 and SYN proteins (85), (5) acupuncture improves neurological deficits and learning memory by upregulating the expression of VEGF and NGFs vascular endothelial factors (86), and (6) acupuncture reduces nerve cell damage by increasing the expression activity of endogenous oxidants such as SOD and GSH-PX proteins (87).

### 4.3. Study strengths and limitations

To the best of our knowledge, this study is the first network meta-analysis to comprehensively assess the efficacy and safety of different acupuncture therapies for PSCI based on currently available evidence. This study was conducted in strict accordance with PRISMA-NMA guidelines. Eight Chinese and English databases were searched to ensure the adequacy of the number of included studies, and references to systematic reviews of relevant topics were also reviewed. We used explicit inclusion and exclusion criteria, data extraction, and risk of bias assessment, and assessed the methodological quality of the included studies using the CONSORT statement guidelines. From the mesh evidence map, we found that only a small number of studies compared different acupuncture therapies directly. Therefore, in the absence of direct evidence, we used the method of network meta-analysis to provide indirect evidence. Finally, according to the nodal split model, it is clear that there is no significant difference between direct or indirect comparisons for each split node (*p* > 0.05), while the Brooks-Gelman-Rubin diagnostic plots for each outcome indicator show that the median and 97.5% values of the contraction factor converge to 1, which indicates that our findings are stable and reliable.

However, there are some limitations of this study that need to be considered. Firstly, acupuncture therapy is a unique non-pharmacological treatment in China, most of the literature related to acupuncture is published in Chinese databases. Therefore, a comprehensive search of Chinese databases is necessary. Unfortunately, most of the included studies were of low quality in terms of experimental design, mainly in the form of a lack of description of allocation concealment, blinding of outcome assessors, and detailed descriptions of prospective plans, which partly contributed to the overall quality of the evidence being rated as low. Seventy-two percent did not achieve the expected reporting rate (>80%) as assessed by the CONSORT statement. In addition, we found a high level of heterogeneity in some comparisons in the results of the paired meta-analysis. Through a review of the literature, we found that this heterogeneity may stem from clinical heterogeneity. Although we compared the same kinds of acupuncture therapies with cognitive training, some factors, such as acupuncture depth, retention time, and acupoint selection, still differed. Differences in the skill level of acupuncture therapists during clinical practice are among the factors that contribute to clinical heterogeneity. Furthermore, given that only thirteen trials in this review were pre-registered, the prospective registration of study protocols before conducting studies should be strongly urged so that others can follow their studies. Moreover, with regard to blinding, it is difficult to blind acupuncture therapists due to the inherent characteristics of acupuncture as a non-pharmacological therapy. However, it is feasible and necessary to blind participants and outcome assessors.

## 5. Conclusion

Based on the available evidence, most acupuncture therapies have positive effects on cognitive function and self-care in daily life in PSCI patients compared to cognitive training. Acupuncture-related therapies may be an effective alternative intervention for the treatment of PSCI. Ophthalmic acupuncture plus cognitive training may be the treatment of choice for improving MMSE scores in PSCI patients. Warm acupuncture plus cognitive training was the preferred therapy for improving MoCA scores, while scalp acupuncture plus body acupuncture was the preferred therapy for improving MBI scores. The methodological quality of the literature included in this study was low and the results should be treated with caution. Future high-quality studies are needed for further validation of our findings.

## Data availability statement

The raw data supporting the conclusions of this article will be made available by the authors, without undue reservation.

## Author contributions

YL, LZ, and FC conceived and designed the study and edited the final manuscript. XL and JH designed the research methodology. MB and XS developed the search strategy and performed data extraction. YL and LZ performed data analysis and wrote the first draft of the manuscript. All authors contributed to the article and approved the submitted version.

## Funding

This study was supported by a grant (The National Natural Science Foundation of China No. 81603684).

## Conflict of interest

The authors declare that the research was conducted in the absence of any commercial or financial relationships that could be construed as a potential conflict of interest.

## Publisher’s note

All claims expressed in this article are solely those of the authors and do not necessarily represent those of their affiliated organizations, or those of the publisher, the editors and the reviewers. Any product that may be evaluated in this article, or claim that may be made by its manufacturer, is not guaranteed or endorsed by the publisher.
