# Supplementary material for: Comparative efficacy and safety of multiple acupuncture therapies for post stroke cognitive impairment: a network meta-analysis of randomized controlled trials
Source: Front Neurol. 2023 Aug 10;14:1218095. doi: 10.3389/fneur.2023.1218095 (PMC10447897; doi:10.3389/fneur.2023.1218095)
Supplement: Supplementary file 2 [file Table_2.DOCX]

**The search strategy for the PubMed database**

| **No** | **Search History** |
| --- | --- |
| #1 | "Stroke"[Mesh] OR "Brain Ischemia"[Mesh] OR "Cerebral Hemorrhage"[Mesh] |
| #2 | (((“Stroke” [Title/Abstract]) OR (Cerebrovascular Accident [Title/Abstract])) OR (Apoplexy [Title/Abstract])) OR (Brain Infarct [Title/Abstract])) OR (Cerebral infarct [Title/Abstract])) OR (Brain stem infarct [Title/Abstract])) OR (Subcortical infarction [Title/Abstract])) OR (Brain venous infarction [Title/Abstract])) OR (Cerebral artery stroke [Title/Abstract])) OR (Cerebral artery infarction [Title/Abstract])) OR (Cerebral circulation infarction [Title/Abstract])) OR (Choroidal artery infarction [Title/Abstract])) OR (Brain ischemia [Title/Abstract])) OR (Ischemic encephalopathy [Title/Abstract]))) |
| #3 | (#1 OR #2) |
| #4 | Cognitive Dysfunction [Mesh] |
| #5 | “Cognitive dysfunction” [Title/Abstract]) OR (cognitive impairment [Title/Abstract])) OR (cognitive decline [Title/Abstract])) OR (mental deterioration [Title/Abstract])) OR (PSCI[Title/Abstract])) OR (aged-related memory disorders [Title/Abstract] OR (Cognitive disorder after stroke [Title/Abstract])) OR (Cognitive impairment after stroke [Title/Abstract]))) |
| #6 | (#4 OR #5) |
| #7 | "Acupuncture"[Mesh] OR "Acupuncture Therapy"[Mesh] |
| #8 | Acupuncture [Title/Abstract]) OR (Acupuncture Therapy [Title/Abstract])) OR (Electro-acupuncture [Title/Abstract])) OR (Ear acupuncture [Title/Abstract])) OR (Auricular acupuncture [Title/Abstract])) OR (Scalp acupuncture [Title/Abstract])) OR (Fire acupuncture [Title/Abstract])) OR (Warm acupuncture [Title/Abstract])) OR (Blood-letting therapy [Title/Abstract])) OR (Moxibustion [Title/Abstract])) OR (Acupoint catgut embedding [Title/Abstract])) OR (Acupoint injection [Title/Abstract])) |
| #9 | (#7 OR #8) |
| #10 | (#3 AND #6 AND #9) |

**The search strategy for the Web of Science database**

| **No** | **Search History** |
| --- | --- |
| #1 | TS= (acupuncture OR acupuncture therapy OR Electroacupuncture OR acupuncture ear OR acupuncture points OR scalp acupuncture OR Ear acupuncture OR Auricular acupuncture OR Fire acupuncture OR Warm acupuncture OR Blood-letting therapy OR Moxibustion OR Acupoint catgut embedding OR Acupoint injection) |
| #2 | TS= (cognitive dysfunction OR cognitive impairment OR cognitive decline OR mental deterioration OR PSCI OR aged-related memory disorders) |
| #3 | TS= (stroke OR stroke, lacunar OR hemorrhagic stroke OR embolic stroke OR thrombotic stroke OR ischemic stroke OR cerebrovascular accident OR cerebrovascular apoplexy OR apoplexy OR lacunar stroke OR lacunar syndrome OR lacunar infarction OR intracerebral hemorrhagic stroke OR intracerebral hemorrhage stroke OR acute ischemic stroke) |
| #4 | (#1 AND #2 AND #3) |

**The search strategy for Embase database**

| **No** | **Search History** |
| --- | --- |
| #1 | acupuncture: ab, ti OR 'acupuncture therapy':ab, ti OR Electroacupuncture: ab, ti OR 'acupuncture ear':ab, ti OR 'acupuncture points':ab, ti OR 'scalp acupuncture':ab, ti OR ' Auricular acupuncture ': ab, ti OR ' Fire acupuncture ': ab, ti OR ' Warm acupuncture ': ab, ti OR ' Blood-letting therapy ': ab, ti OR ' Moxibustion ': ab, ti OR ' Acupoint catgut embedding ': ab, ti OR ' Acupoint injection ': ab, ti |
| #2 | 'cognitive dysfunction':ab, ti OR 'cognitive impairment':ab, ti OR 'cognitive decline':ab, ti OR 'mental deterioration':ab, ti OR PSCI: ab, ti OR 'aged-related memory disorders':ab, ti |
| #3 | stroke:ab,ti OR stroke,lacunar:ab,ti OR 'hemorrhagic stroke':ab,ti OR 'embolic stroke':ab,ti OR 'thrombotic stroke':ab,ti OR 'ischemic stroke':ab,ti OR 'cerebrovascular accident':ab,ti OR 'cerebrovascular apoplexy':ab,ti OR apoplexy:ab,ti OR 'lacunar stroke':ab,ti OR 'lacunar syndrome':ab,ti OR 'lacunar infarction':ab,ti OR 'intracerebral hemorrhagic stroke':ab,ti OR 'intracerebral hemorrhage stroke':ab,ti OR 'acute ischemic stroke':ab,ti |
| #4 | (#1 AND #2 AND #3) |

**The search strategy for the Cochrane Library database**

| **No** | **Search History** |
| --- | --- |
| #1 | acupuncture OR acupuncture therapy OR Electroacupuncture OR acupuncture ear OR acupuncture points OR scalp acupuncture OR Ear acupuncture OR Auricular acupuncture OR Fire acupuncture OR Warm acupuncture OR Blood-letting therapy OR Moxibustion OR Acupoint catgut embedding OR Acupoint injection: ti, ab,kw |
| #2 | cognitive dysfunction OR cognitive impairment OR cognitive decline OR mental deterioration OR PSCI OR aged-related memory disorders): ti, ab,kw |
| #3 | stroke OR stroke, lacunar OR hemorrhagic stroke OR embolic stroke OR thrombotic stroke OR ischemic stroke OR cerebrovascular accident OR cerebrovascular apoplexy OR apoplexy OR lacunar stroke OR lacunar syndrome OR lacunar infarction OR intracerebral hemorrhagic stroke OR intracerebral hemorrhage stroke OR acute ischemic stroke): ti, ab,kw |
| #4 | (#1 AND #2 AND #3) |

**The search strategy for China National Knowledge Infrastructure (CNKI) database**

| **No** | **Search History** |
| --- | --- |
| #1 | 主题：针灸疗法+针灸疗法+毫针（精确） |
| #2 | 篇摘要：针刺疗法+针灸疗法+毫针+体针+头针+电针+针灸+针刺（精确） |
| #3 | (#1 OR #2) |
| #4 | 主题：中风+卒中+脑血管障碍（精确） |
| #5 | 篇摘要：中风+卒中+脑血管障碍+脑梗塞+脑梗死+脑出血+脑卒中+脑栓塞+脑血管病+脑血管意外（精确） |
| #6 | (#4 OR #5) |
| #7 | 主题：认知障碍（精确） |
| #8 | 篇关摘：认知障碍+认知功能障碍+认知损伤+认知损害+神经行为障碍（精确） |
| #9 | (#7 OR #8) |
| #10 | (#3 AND #6 AND #9) |

**The search strategy for the Chinese Biological Medicine Database**

| **No** | **Search History** |
| --- | --- |
| #1 | "针灸疗法"[不加权:扩展] OR "针刺疗法"[不加权:扩展] OR "毫针"[不加权:扩展] |
| #2 | "针灸疗法"[全部字段: 智能] OR "针刺疗法"[全部字段: 智能] OR "毫针"[全部字段: 智能] OR "电针"[全部字段: 智能] OR "头针"[全部字段: 智能] OR "耳针"[全部字段: 智能] OR "体针"[全部字段: 智能] OR "针灸"[全部字段: 智能] OR "针刺"[全部字段: 智能] |
| #3 | (#1) OR (#2) |
| #4 | "卒中"[不加权:扩展] OR "中风"[不加权:扩展] OR "脑血管障碍"[不加权:扩展] |
| #5 | "卒中"[全部字段: 智能] OR "中风"[全部字段: 智能] OR "脑栓塞"[全部字段: 智能] OR "脑卒中"[全部字段: 智能] OR "脑梗赛"[全部字段: 智能] OR "脑梗死"[全部字段: 智能] OR "脑出血"[全部字段: 智能] OR "脑血管意外"[全部字段: 智能] OR "脑血管病"[全部字段: 智能] |
| #6 | (#4) OR (#5) |
| #7 | "认知障碍"[不加权:扩展] |
| #8 | "认知障碍"[全部字段: 智能] OR "认知功能障碍"[全部字段: 智能] OR "认知损害"[全部字段: 智能] OR "认知损伤"[全部字段: 智能] OR "神经行为障碍"[全部字段: 智能] |
| #9 | (#7 OR #8) |
| #10 | (#3 AND #6 AND #9) |

**The search strategy for the Chinese Science and Technology Periodical Database (VIP)**

| **No** | **Search History** |
| --- | --- |
| #1 | 题名或关键词=针刺疗法 OR 针灸疗法 OR 毫针 OR 电针 OR 体针 OR 头针 OR 耳针 OR 针刺 OR 针灸 |
| #2 | 题名或关键词=卒中 OR 中风 OR 脑血管障碍 OR 脑卒中 OR 脑梗死 OR 脑梗塞 OR 脑出血 OR 脑栓塞 OR 脑血管病 OR 脑血管病意外 |
| #3 | 题名或关键词=认知障碍 OR 认知功能障碍 OR 认知损害 OR 认知损伤 OR 神经行为障碍 |
| #4 | (#1 AND #2 AND #3) |

**The search strategy for Wan Fang Database**

| **No** | **Search History** |
| --- | --- |
| #1 | 题名或关键词=针刺疗法 OR 针灸疗法 OR 毫针 OR 电针 OR 体针 OR 头针 OR 耳针 OR 针刺 OR 针灸 |
| #2 | 题名或关键词=卒中 OR 中风 OR 脑血管障碍 OR 脑卒中 OR 脑梗死 OR 脑梗塞 OR 脑出血 OR 脑栓塞 OR 脑血管病 OR 脑血管病意外 |
| #3 | 题名或关键词=认知障碍 OR 认知功能障碍 OR 认知损害 OR 认知损伤 OR 神经行为障碍 |
| #4 | (#1 AND #2 AND #3) |
