# Supplementary material for: Comparative efficacy and safety of multiple acupuncture therapies for post stroke cognitive impairment: a network meta-analysis of randomized controlled trials
Source: Front Neurol. 2023 Aug 10;14:1218095. doi: 10.3389/fneur.2023.1218095 (PMC10447897; doi:10.3389/fneur.2023.1218095)
Supplement: Supplementary file 4 [file Data_Sheet_1.docx]

**Supplementary Figure**

**Comparative efficacy and safety of multiple acupuncture therapies for post-stroke cognitive impairment: a network meta-analysis of randomized controlled trials**

**Yang Liu^1,2^, Lu Zhao^1,2^, Fuyan Chen^1,2,*^, Xingping Li^1,2^, Jiangqin Han^1,2^, Xiaowei Sun^1,2^, Mingtong Bian^1,2^**

^1^ Department of Acupuncture. First Teaching Hospital of Tianjin University of Traditional Chinese Medicine, Anshanxi Road, Nankai District, Tianjin 300193, China

^2^ National Clinical Research Center for Chinese Medicine Acupuncture and Moxibustion, Tianjin 300193, China

- **Figure S1** The result of the risk of bias assessment 3
- **Figure S2** The result of the inconsistency test 6
- **Figure S3** The results of Brooks-Gelman-Rubin diagnostic Plots 9
- **Figure S4** The results of trajectory and density Plots 13

**Supplementary Figure S1 The result of the risk of bias assessment**

| **Study ID** | | **D1** | **D2** | **D3** | | **D4** | **D5** | **Overall** |
| --- | --- | --- | --- | --- | --- | --- | --- | --- |
| 01-Bao 2021 | |  |  |  | |  |  |  |
| 02 Jiang 2016 | |  |  |  | |  |  |  |
| 03-Jian Xiong 2020 | |  |  |  | |  |  |  |
| 04-Bai jing 2012 | |  |  |  | |  |  |  |
| 05-Pu Yuan 2018 | |  |  |  | |  |  |  |
| 06-Cai 2020 | |  |  |  | |  |  |  |
| 07-Zeng 2018 | |  |  |  | |  |  |  |
| 08-Chen 2020 | |  |  |  | |  |  |  |
| 09-Liu 2013 | |  |  |  | |  |  |  |
| 10-Chen 2020 | |  |  |  | |  |  |  |
| 11-Ding 2016 | |  |  |  | |  |  |  |
| 12-Du 2019 | |  |  |  | |  |  |  |
| 13-Duan 2021 | |  |  |  | |  |  |  |
| 14-Feng 2014 | |  |  |  | |  |  |  |
| 15-Han 2021 | |  |  |  | |  |  |  |
| 16-Hu 2019 | |  |  |  | |  |  |  |
| 17-Niu 2021 | |  |  |  | |  |  |  |
| 18-Kong 2021 | |  |  |  | |  |  |  |
| 19-Leng 2020 | |  |  |  | |  |  |  |
| 20-Chen 2016 | |  |  |  | |  |  |  |
| 21-Li 2017 | |  |  |  | |  |  |  |
| 22-Lin 2020 | |  |  |  | |  |  |  |
| 23-Lin 2014 | |  |  |  | |  |  |  |
| 24-Qian 2018 | |  |  |  | |  |  |  |
| 25-Feng 2015 | |  |  |  | |  |  |  |
| 26-Song 2020 | |  |  |  | |  |  |  |
| 27-Sun 2019 | |  |  |  | |  |  |  |
| 28-Tan 2020 | |  |  |  | |  |  |  |
| 29-Ge 2016 | |  |  |  | |  |  |  |
| 30-Tian 2021 | |  |  |  | |  |  |  |
| 31-Wang 2015 | |  |  |  | |  |  |  |
| 32-Wang 2021 | |  |  |  | |  |  |  |
| 33-Wang 2021 | |  |  |  | |  |  |  |
| 34-Wang 2017 | |  |  |  | |  |  |  |
| 35-Wang 2015 | |  |  |  | |  |  |  |
| 36-Wang 2018 | |  |  |  | |  |  |  |
| 37-Wang 2011 | |  |  |  | |  |  |  |
| 38-Lei 2021 | |  |  |  | |  |  |  |
| 39-Wang 2018 | |  |  |  | |  |  |  |
| 40-Wang 2019 | |  |  |  | |  |  |  |
| 41-Zhang 2021 | |  |  |  | |  |  |  |
| 42-Wei 2019 | |  |  |  | |  |  |  |
| 43-Zhao 2021 | |  |  |  | |  |  |  |
| 44-Xu 2022 | |  |  |  | |  |  |  |
| 45-Zheng 2021 | |  |  |  | |  |  |  |
| 46-Yan 2022 | |  |  |  | |  |  |  |
| 47-Yang 2018 | |  |  |  | |  |  |  |
| 48-Yang 2020 | |  |  |  | |  |  |  |
| 49-Zhou 2021 | |  |  |  | |  |  |  |
| 50-Yang 2019 | |  |  |  | |  |  |  |
| 51-Yao 2019 | |  |  |  | |  |  |  |
| 52-Yao 2020 | |  |  |  | |  |  |  |
| 53-Yu 2021 | |  |  |  | |  |  |  |
| 54-Zhan 2016 | |  |  |  | |  |  |  |
| 55-Zhang 2020 | |  |  |  | |  |  |  |
| 56-Zhang 2018 | |  |  |  | |  |  |  |
| 57-Zhang 2019 | |  |  |  | |  |  |  |
| 58-Zheng 2019 | |  |  |  | |  |  |  |
| 59-Zhou 2022 | |  |  |  | |  |  |  |
| 60-Zhuo 2021 | |  |  |  | |  |  |  |
| 61-Zhou 2020 | |  |  |  | |  |  |  |
| 62-Zhu 2014 | |  |  |  | |  |  |  |
|  |  | | | |  |  |  |  |

|  | Low risk |
| --- | --- |
|  | Some concerns |
|  | High risk |
| D1 | Randomisation process |
| D2 | Deviations from the intended interventions |
| D3 | Missing outcome data |
| D4 | Measurement of the outcome |
| D5 | Selection of the reported result |

**Supplementary Figure S2 The result of the inconsistency test**


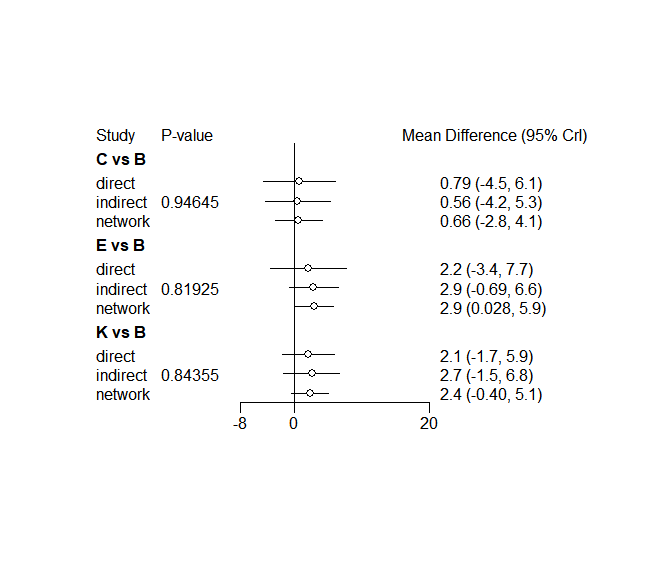

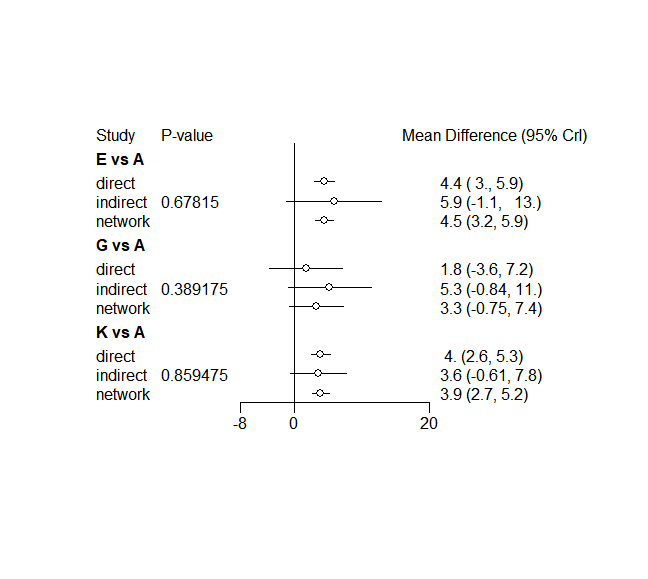

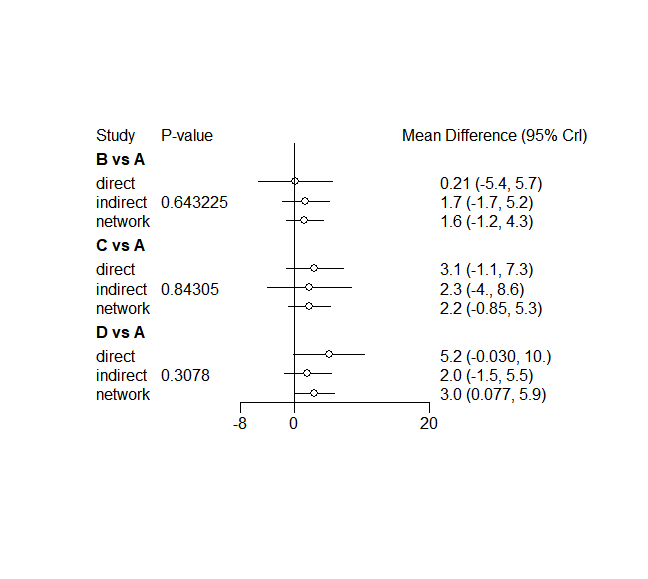
**MMSE**

**
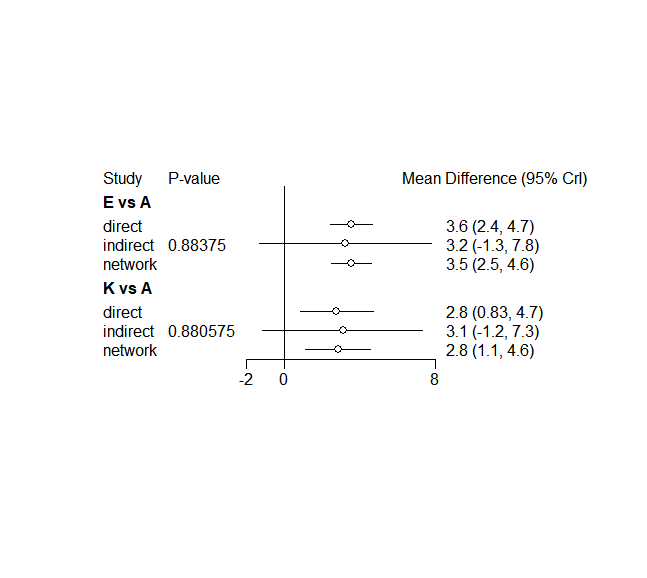

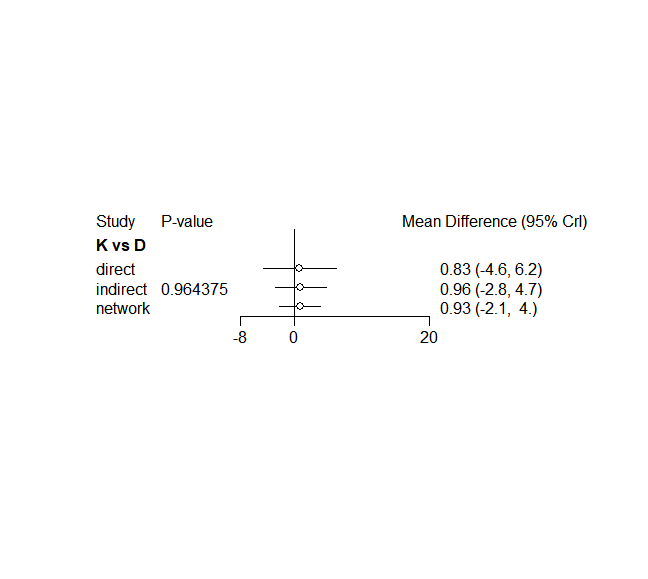

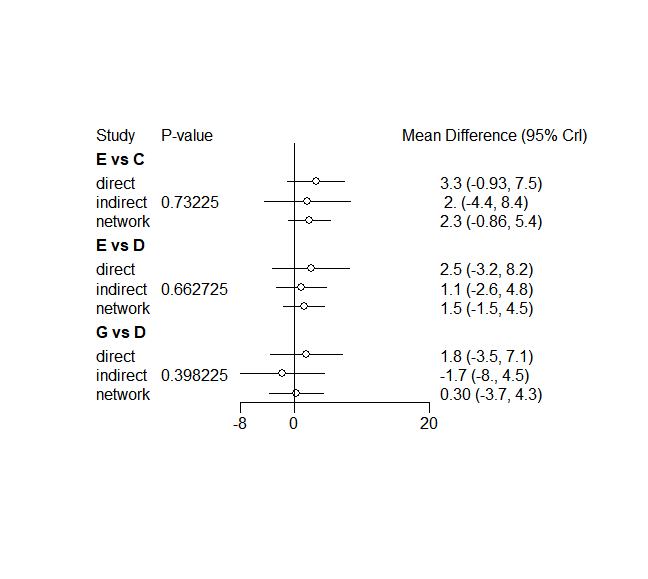
MoCA**

**
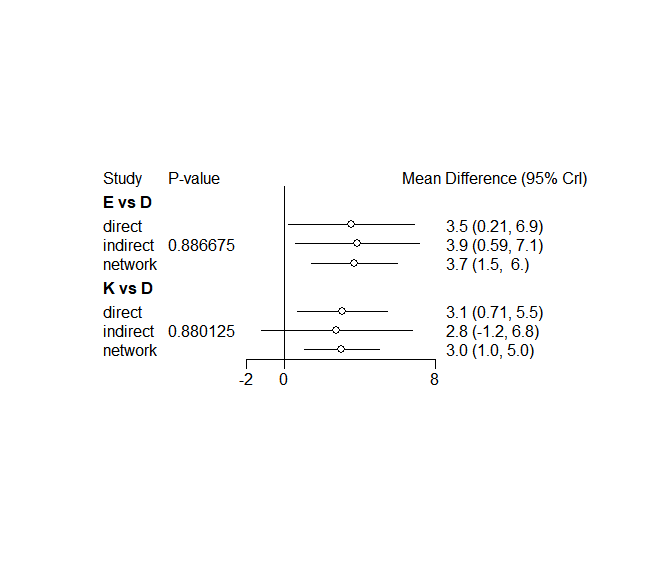

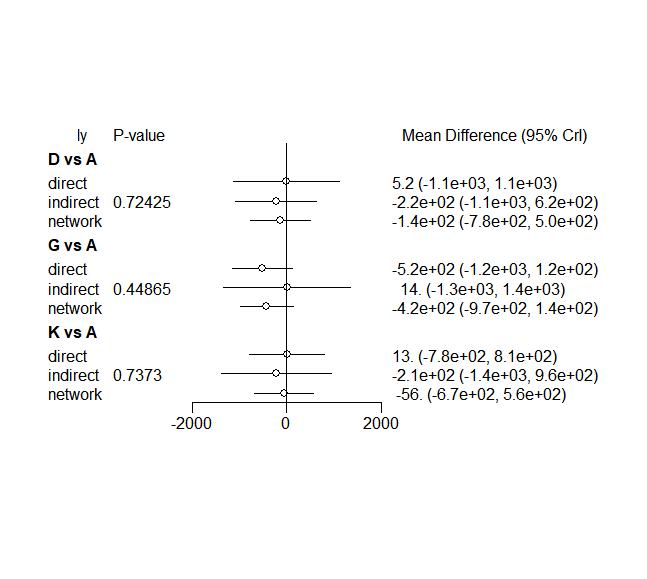

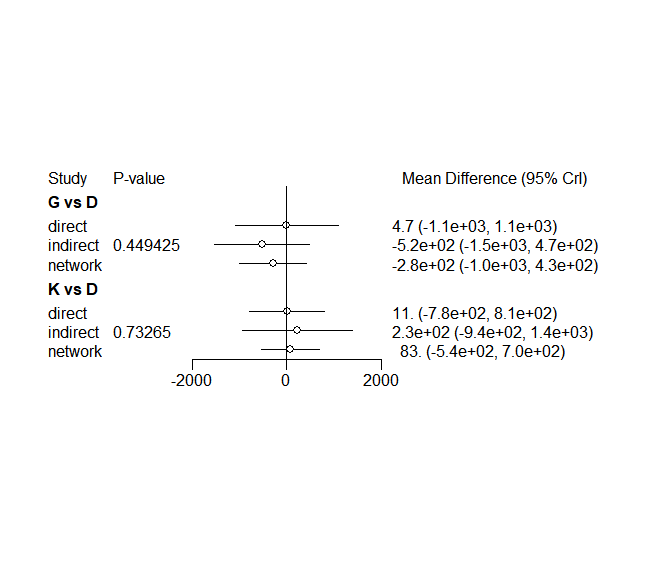
MBI**

**Supplementary Figure S3 The result of Brooks-Gelman-Rubin diagnostic Plots**

**
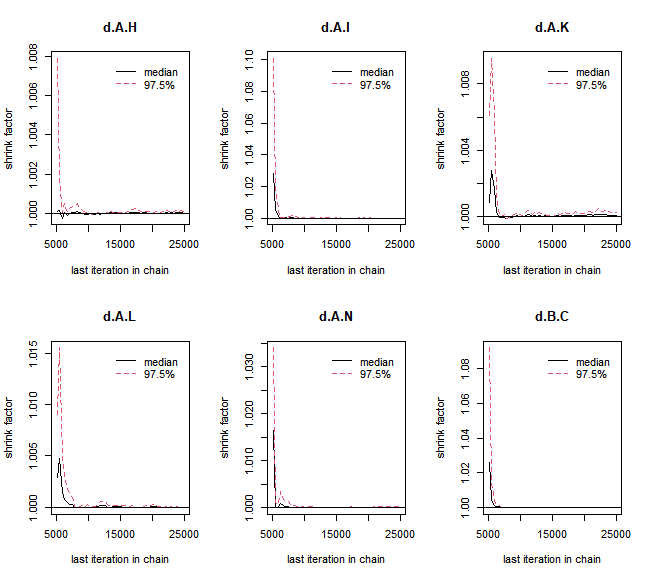

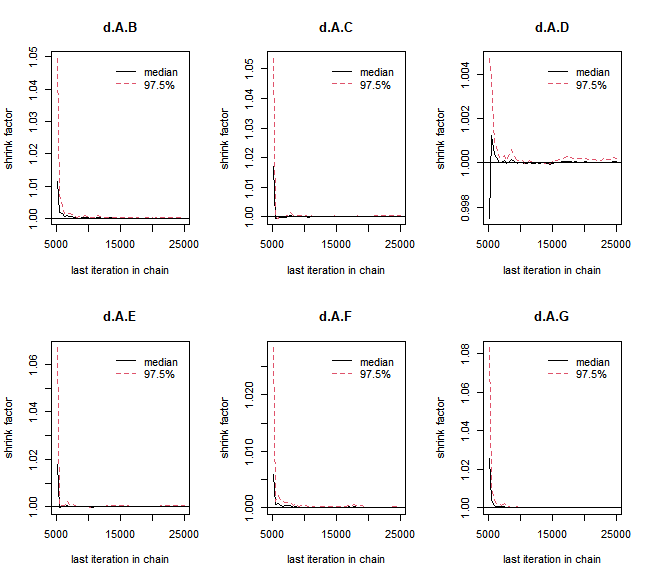
MMSE:**

**
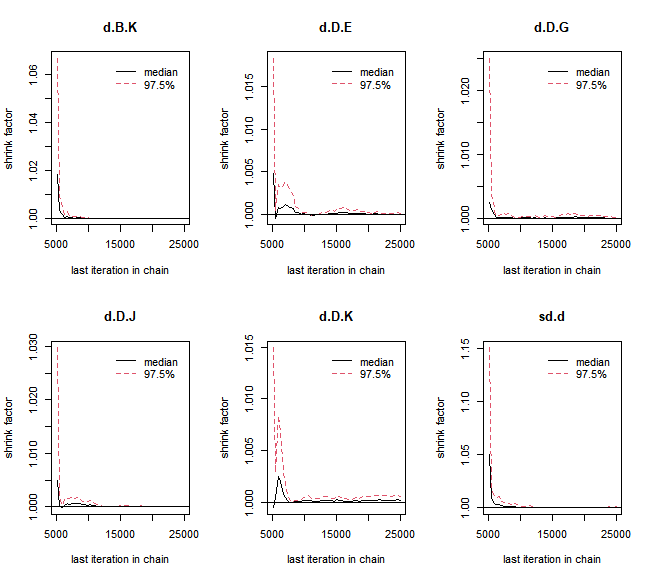
**

**
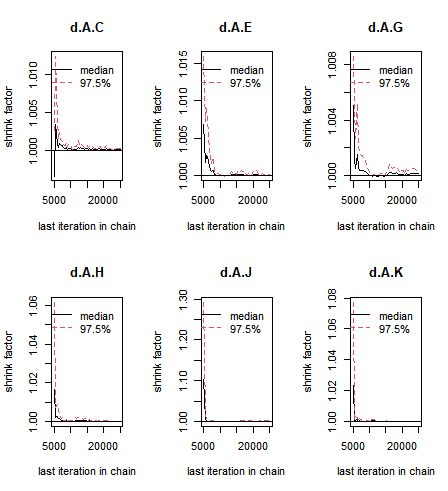
MoCA:**

**
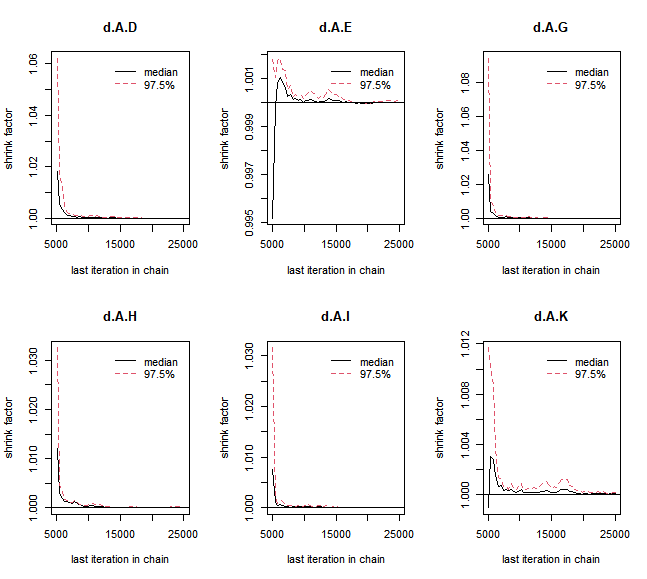

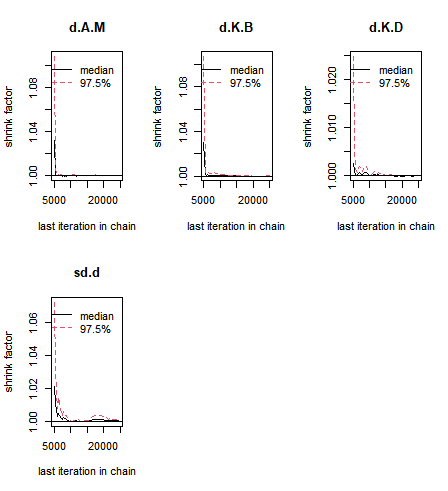
MBI**

**
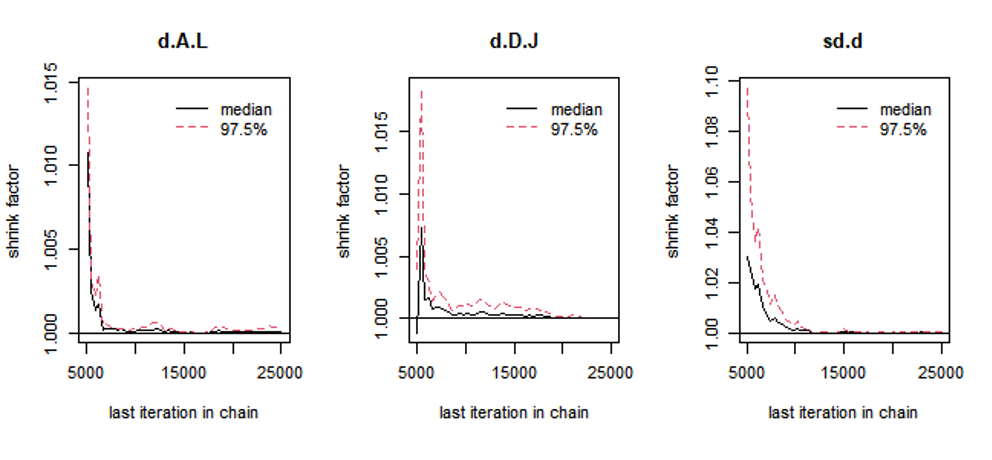
**

A: Cognitive training; B: Body acupuncture; C: Scalp acupuncture; D: Body acupuncture plus cognitive training; E: Scalp acupuncture plus cognitive training; F: Ophthalmic acupuncture plus cognitive training; G: Warm acupuncture plus cognitive training; H: Electro-acupuncture plus cognitive training; I: Auricular bloodletting plus cognitive training; J: Abdominal acupuncture plus cognitive training; K: Scalp acupuncture plus body acupuncture; L: Abdominal acupuncture plus body acupuncture; M: Warm acupuncture plus scalp acupuncture; N: Scalp acupuncture plus auricular bloodletting

**Supplementary Figure S4 The result of trajectory and density Plots**

**
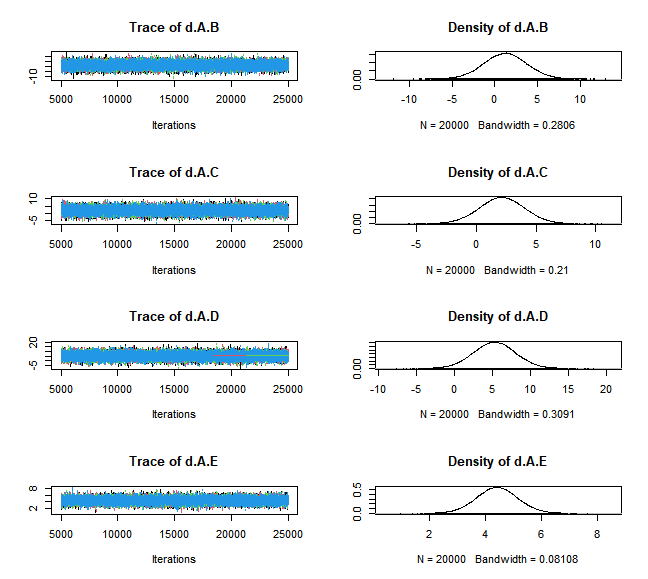
MMSE:**

**
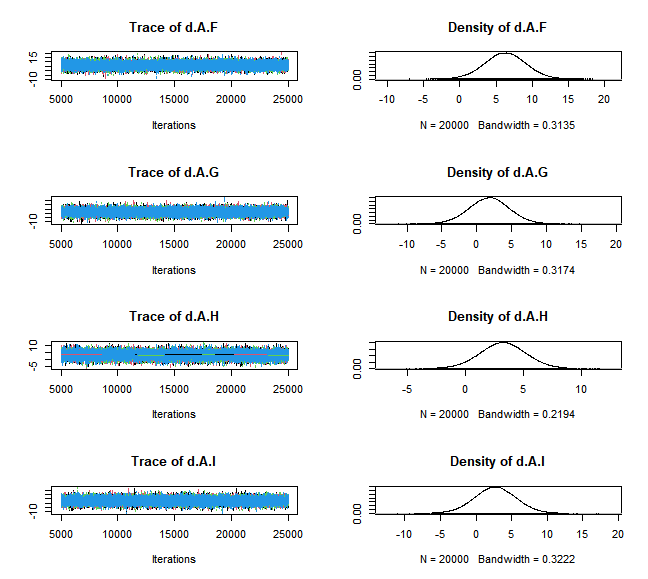
**

**
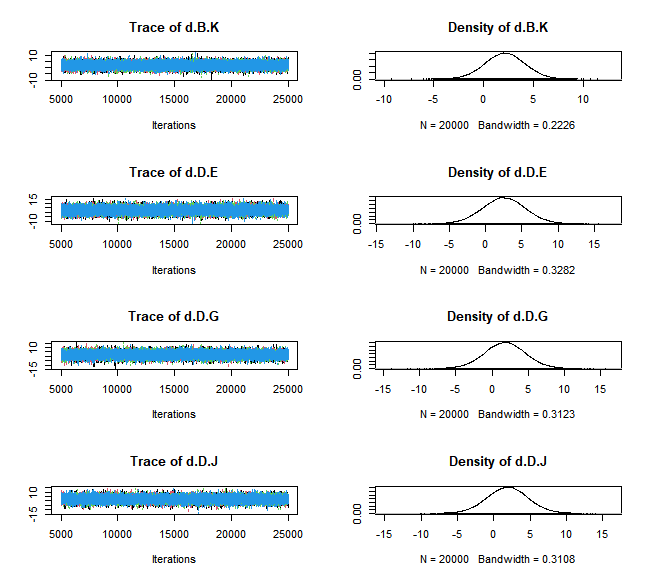

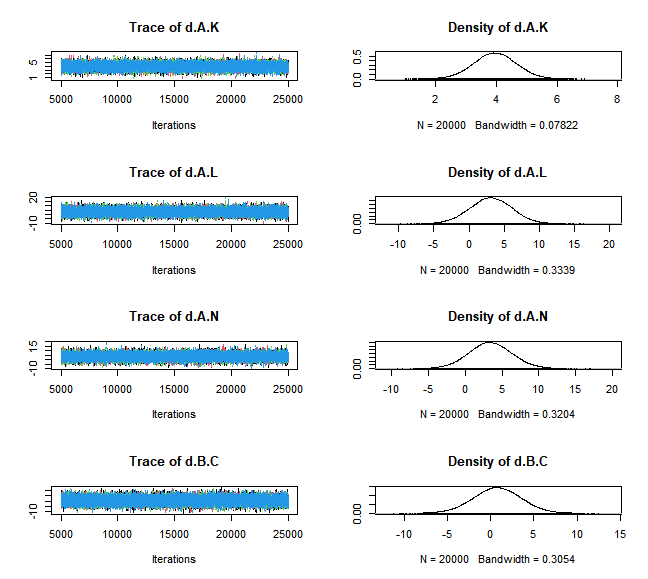
**

**
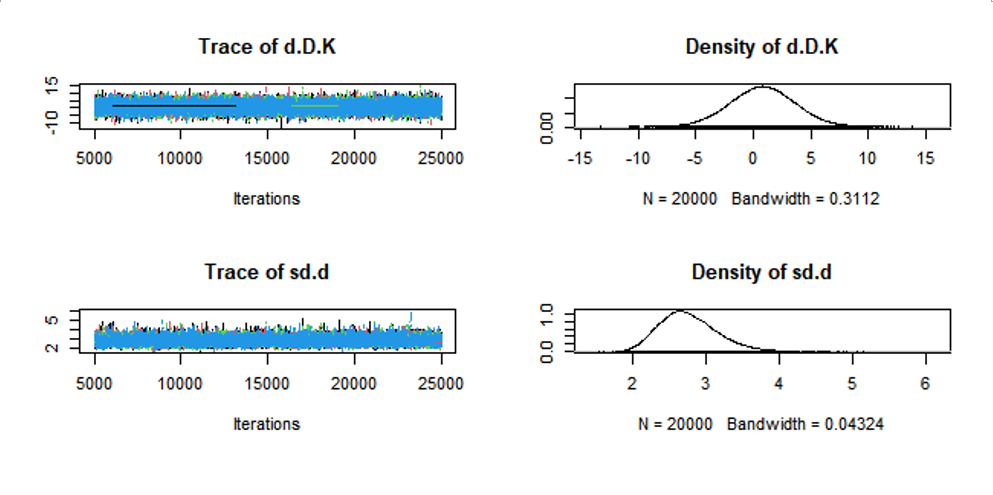
**

**
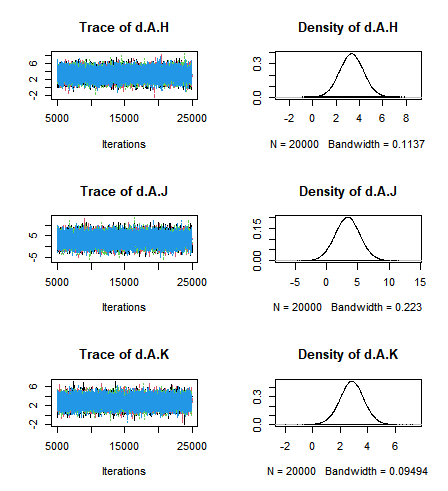

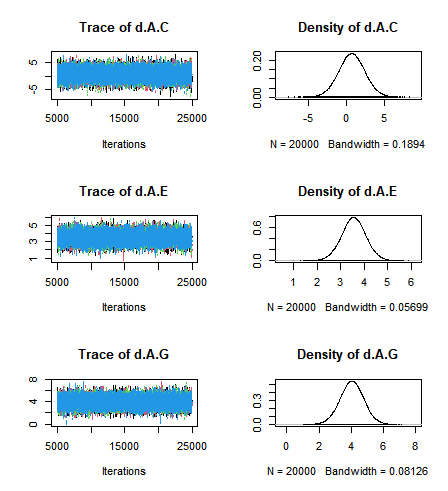
MoCA:**

**
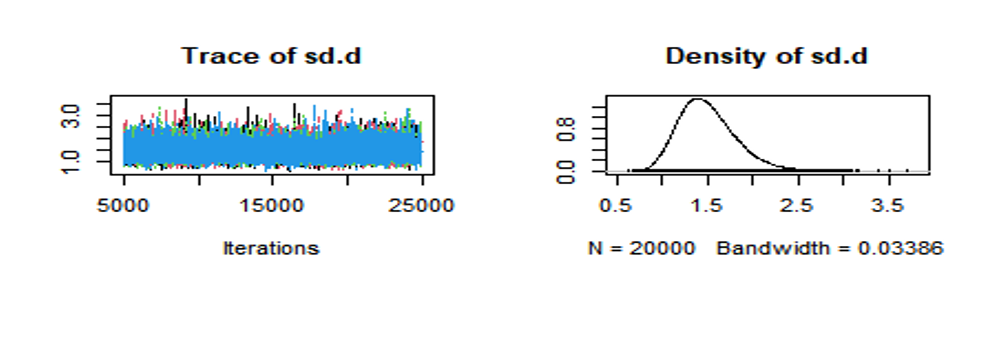

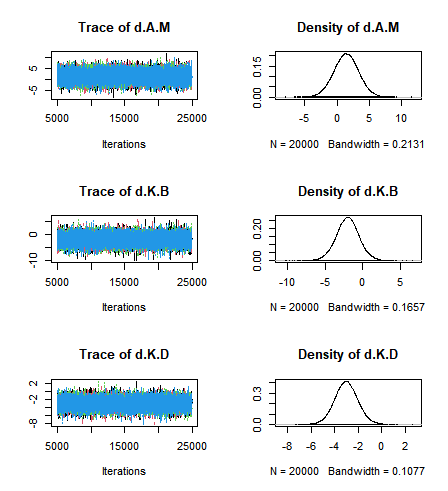
**

**
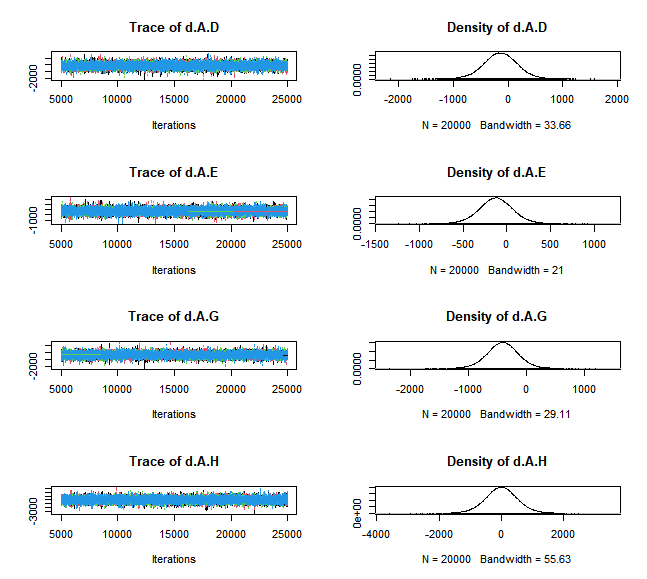
MBI**

**
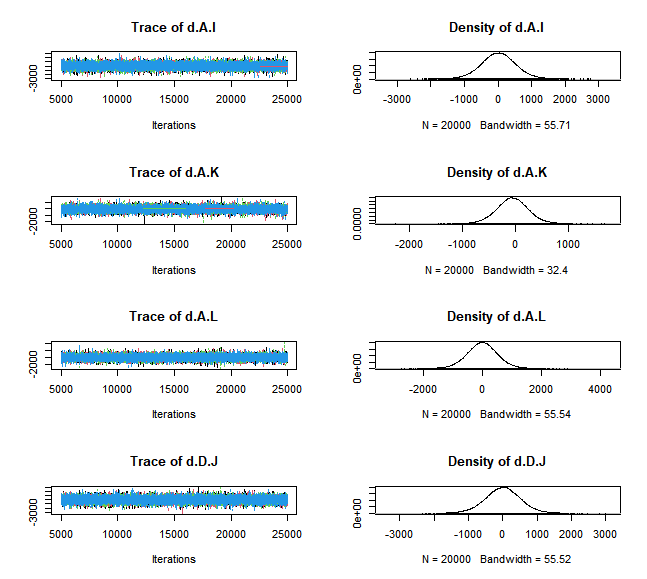
**

**
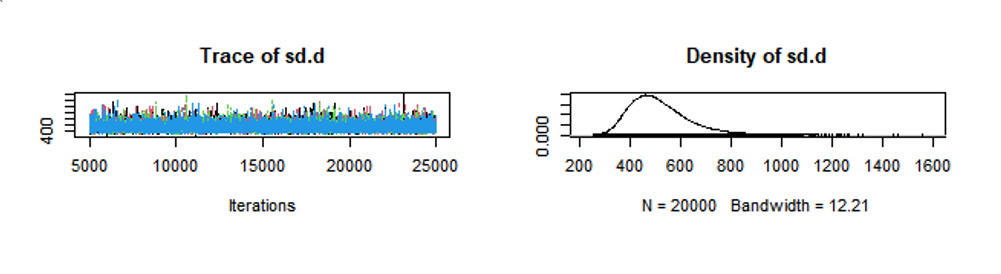
**

Treatment: A: Conventional training; B: Conventional acupuncture; C: Scalp acupuncture; D: Scalp acupuncture plus Conventional training; E: ophthalmic acupuncture plus cognitive training; F: warm acupuncture plus cognitive training; G: electro-acupuncture plus cognitive training; H: auricular bloodletting plus cognitive training; I: abdominal acupuncture plus cognitive training; J: conventional acupuncture plus cognitive training; K: abdominal acupuncture plus conventional acupuncture; L: scalp acupuncture plus conventional acupuncture; M: . scalp acupuncture plus auricular bloodletting
