# Supplementary material for: Comparative efficacy and safety of multiple acupuncture therapies for post stroke cognitive impairment: a network meta-analysis of randomized controlled trials
Source: Front Neurol. 2023 Aug 10;14:1218095. doi: 10.3389/fneur.2023.1218095 (PMC10447897; doi:10.3389/fneur.2023.1218095)
Supplement: Supplementary file 5 [file Data_Sheet_2.docx]

**Supplementary Table**

**Comparative efficacy and safety of multiple acupuncture therapies for post-stroke cognitive impairment: a network meta-analysis of randomized controlled trials**

**Yang Liu^1,2^, Lu Zhao^1,2^, Fuyan Chen^1,2,*^, Xingping Li^1,2^, Jiangqin Han^1,2^, Xiaowei Sun^1,2^, Mingtong Bian^1,2^**

^1^ Department of Acupuncture. First Teaching Hospital of Tianjin University of Traditional Chinese Medicine, Anshanxi Road, Nankai District, Tianjin 300193, China

^2^ National Clinical Research Center for Chinese Medicine Acupuncture and Moxibustion, Tianjin 300193, China

- **Table S1** The results of the closed-loop inconsistency test 3
- **Table S2** The value of SUCRA for each outcome 4
- **Table S3** The summary of Adverse Effects 5
- **Table S4** The results of Evidence Assessment 5

**Supplementary Table S1 The results of the closed-loop inconsistency test**

| **Loop** | **IF** | **seIF** | **Z_value** | **P_value** | **95%CI** | **Loop_Heterog_tau2** |
| --- | --- | --- | --- | --- | --- | --- |
| **MMSE** | | | | | | |
| A-D-G | 5.21 | 1.09 | 4.762 | 0.000 | **(0.00, 7.35)** | 0.000 |
| A-D-E | 3.316 | 4.519 | 0.734 | 0.463 | **(0.00, 12.17)** | 7.008 |
| A-B-E | 2.224 | 4.618 | 0.482 | 0.630 | **(0.00, 11.27)** | 7.370 |
| A-C-E | 2.118 | 3.342 | 0.634 | 0.526 | **(0.00, 8.67)** | 6.759 |
| A-D-K | 2.093 | 3.616 | 0.579 | 0.563 | **(0.00, 9.18)** | 5.020 |
| A-B-C | 2.084 | 1.310 | 1.591 | 0.112 | **(0.00, 4.65)** | 0.000 |
| B-C-E | 1.829 | 1.303 | 1.403 | 0.161 | **(0.00, 4.38)** | 0.000 |
| A-B-E | 1.631 | 3.136 | 0.520 | 0.603 | **(0.00, 7.78)** | 4.784 |
| B-D-E-K | 1.499 | 1.624 | 0.923 | 0.356 | **(0.00, 4.68)** | 0.000 |
| **MoCA** | | | | | | |
| A-C-E | 1.429 | 2.853 | 0.501 | 0.616 | **(0.00, 7.02)** | 2.410 |
| A-D-E-I | 0.331 | 2.185 | 0.152 | 0.879 | **(0.00, 4.61)** | 2.100 |
| **MBI** | | | | | | |
| A-B-H | 2.956 | 6.087 | 0.486 | 0.627 | **(0.00, 14.89)** | 13.219 |
| A-B-D | 2.322 | 2.257 | 1.029 | 0.304 | **(0.00, 6.75)** | 0.000 |

A: Cognitive training; B: Body acupuncture; C: Scalp acupuncture; D: Body acupuncture plus cognitive training; E: Scalp acupuncture plus cognitive training; F: Ophthalmic acupuncture plus cognitive training; G: Warm acupuncture plus cognitive training; H: Electro-acupuncture plus cognitive training; I: Auricular bloodletting plus cognitive training; J: Abdominal acupuncture plus cognitive training; K: Scalp acupuncture plus body acupuncture; L: Abdominal acupuncture plus body acupuncture; M: Warm acupuncture plus scalp acupuncture; N: Scalp acupuncture plus auricular bloodletting; MMSE: The Minimum Mental State Examination scale; MoCA: The Montreal Cognitive Assessment Scale; MBI: The Modified Barthel Index scale

**Supplementary Table S2 The Value of SUCRA for each outcome**

| **Treatment** | **MMSE** | | | **MoCA** | | | **MBI** | | |
| --- | --- | --- | --- | --- | --- | --- | --- | --- | --- |
|  | SUCRA | Pr Best | MeanRank | SUCRA | Pr Best | MeanRank | SUCRA | Pr Best | MeanRank |
| A | 6.2 | 0.0 | 12.3 | 16.4 | 0.0 | 8.5 | 13.4 | 0.0 | 7.9 |
| B | 26.0 | 0.0 | 9.9 | 30.7 | 1.2 | 7.2 | - | - | - |
| C | 36.0 | 0.4 | 8.7 | 29.0 | 1.1 | 7.4 | - | - | - |
| D | 46.6 | 0.3 | 7.4 | 15.2 | 0.0 | 8.6 | 36.7 | 0.0 | 6.1 |
| E | 73.7 | 3.5 | 4.2 | 77.3 | 10.0 | 3.0 | 72.6 | 8.5 | 3.2 |
| F | ***79.7*** | 37.5 | 3.4 | **-** | **-** | - | - | - | - |
| G | 51.7 | 4.1 | 6.8 | **86.5** | 35.9 | 2.2 | 62.0 | 3.4 | 4.0 |
| H | 51.2 | 3.8 | 6.9 | 72.1 | 14.1 | 3.5 | 19.5 | 0.3 | 7.4 |
| I | 44.4 | 6.4 | 7.7 | - | - | - | 75.4 | 32.3 | 3.0 |
| J | 69.5 | 24.9 | 4.7 | 69.7 | 30.0 | 3.7 | 61.2 | 14.1 | 4.1 |
| K | 62.8 | 0.7 | 5.5 | 62.8 | 3.0 | 4.3 | **87.5** | 40.8 | 2.0 |
| L | 49.0 | 8.6 | 7.1 | - | - | - | 21.7 | 0.6 | 7.3 |
| M | - | - | - | - | - | - | - | - | - |
| N | 53.2 | 9.8 | 6.6 | 40.2 | 4.7 | 6.4 | - | - | - |

A: Cognitive training; B: Body acupuncture; C: Scalp acupuncture; D: Body acupuncture plus cognitive training; E: Scalp acupuncture plus cognitive training; F: Ophthalmic acupuncture plus cognitive training; G: Warm acupuncture plus cognitive training; H: Electro-acupuncture plus cognitive training; I: Auricular bloodletting plus cognitive training; J: Abdominal acupuncture plus cognitive training; K: Scalp acupuncture plus body acupuncture; L: Abdominal acupuncture plus body acupuncture; M: Warm acupuncture plus scalp acupuncture; N: Scalp acupuncture plus auricular bloodletting; MMSE: The Minimum Mental State Examination scale; MoCA: The Montreal Cognitive Assessment Scale; MBI: The Modified Barthel Index scale

**Supplementary Table S3** The summary of Adverse Effects

| **Study** | **Intervention** | **Adverse Effects** |
| --- | --- | --- |
| Bao 2021 | SA+BA | No adverse effects |
| Jiang 2016 | SA+CT | No adverse effects |
| Lin 2020 | SA+BA | No adverse effects |
| Sun 2019 | SA+BA | One case of dizziness, one case of weakness and one case of pallor in each of the experimental and control groups |
| Zheng 2021 | SA+BA | Two cases in the experimental group felt pain at the acupuncture site and one case complained of dizziness, while two cases in the control group complained of panic |
| Yao 2019 | EA+CT | Three cases of subcutaneous hematoma were observed in the experimental group (resolved without treatment) |

SA: Scalp acupuncture; BA: Body acupuncture; EA: Electro-acupuncture; CT: Cognitive training

**Supplementary Table S4** The results of Evidence Assessment

| **Comparison** | **Risk of bias** | **Inconsistency** | **Indirectness** | **Imprecision** | **Publication Bias** | **GRADE** |
| --- | --- | --- | --- | --- | --- | --- |
| **MMSE** | | | | | | |
| K-A | Serious | Serious | Not serious | Not serious | Not serious | LOW |
| E-A | Serious | Serious | Not serious | Not serious | Not serious | LOW |
| D-A | Serious | Not serious | Not serious | Serious | Not serious | LOW |
| F-A | Serious | Not serious | Not serious | Serious | Not serious | LOW |
| G-A | Serious | Not serious | Not serious | Serious | Not serious | LOW |
| I-A | Serious | Not serious | Not serious | Serious | Not serious | LOW |
| N-A | Serious | Not serious | Not serious | Serious | Not serious | LOW |
| E-B | Serious | Not serious | Not serious | Serious | Not serious | LOW |
| E-C | Serious | Not serious | Not serious | Serious | Not serious | LOW |
| E-D | Serious | Not serious | Not serious | Serious | Not serious | LOW |
| G-D | Serious | Not serious | Not serious | Serious | Not serious | LOW |
| K-B | Serious | Not serious | Not serious | Serious | Not serious | LOW |
| C-A | Serious | Not serious | Not serious | Serious | Not serious | LOW |
| C-B | Serious | Not serious | Not serious | Serious | Not serious | LOW |
| B-A | Serious | Not serious | Not serious | Very serious | Not serious | VERY LOW |
| H-A | Serious | Serious | Not serious | Very serious | Not serious | VERY LOW |
| L-A | Serious | Not serious | Not serious | Very serious | Not serious | VERY LOW |
| J-D | Serious | Not serious | Not serious | Very serious | Not serious | VERY LOW |
| K-D | Serious | Not serious | Not serious | Very serious | Not serious | VERY LOW |
| **MoCA** | | | | | | |
| E-A | Serious | Serious | Not serious | Not serious | Not serious | LOW |
| K-A | Serious | Not serious | Not serious | Not serious | Not serious | Moderate |
| G-A | Serious | Serious | Not serious | Not serious | Not serious | LOW |
| H-A | Serious | Not serious | Not serious | Serious | Not serious | LOW |
| J-A | Serious | Not serious | Not serious | Serious | Not serious | LOW |
| E-D | Serious | Not serious | Not serious | Serious | Not serious | LOW |
| K-D | Serious | Serious | Not serious | Serious | Not serious | VERY LOW |
| K-B | Serious | Not serious | Not serious | Serious | Not serious | LOW |
| E-C | Serious | Not serious | Not serious | Very serious | Not serious | VERY LOW |
| C-A | Serious | Not serious | Not serious | Very serious | Not serious | VERY LOW |
| M-A | Serious | Not serious | Not serious | Very serious | Not serious | VERY LOW |
| **MBI** | | | | | | |
| D-A | Serious | Not serious | Not serious | Serious | Not serious | LOW |
| E-A | Serious | Serious | Not serious | Not serious | Not serious | LOW |
| G-A | Serious | Serious | Not serious | Serious | Not serious | VERY LOW |
| K-A | Serious | Not serious | Not serious | Serious | Not serious | LOW |
| H-A | Serious | Not serious | Not serious | Serious | Not serious | LOW |
| I-A | Serious | Not serious | Not serious | Serious | Not serious | LOW |
| J-D | Serious | Not serious | Not serious | Serious | Not serious | LOW |
| K-D | Serious | Serious | Not serious | Serious | Not serious | VERY LOW |
| G-D | Serious | Not serious | Not serious | Serious | Not serious | LOW |
| L-A | Serious | Not serious | Not serious | Very serious | Not serious | VERY LOW |

A: Cognitive training; B: Body acupuncture; C: Scalp acupuncture; D: Body acupuncture plus cognitive training; E: Scalp acupuncture plus cognitive training; F: Ophthalmic acupuncture plus cognitive training; G: Warm acupuncture plus cognitive training; H: Electro-acupuncture plus cognitive training; I: Auricular bloodletting plus cognitive training; J: Abdominal acupuncture plus cognitive training; K: Scalp acupuncture plus body acupuncture; L: Abdominal acupuncture plus body acupuncture; M: Warm acupuncture plus scalp acupuncture; N: Scalp acupuncture plus auricular bloodletting; MMSE: The Minimum Mental State Examination scale; MoCA: The Montreal Cognitive Assessment Scale; MBI: The Modified Barthel Index scale
